# Impact heat driven volatile redistribution at Occator crater on Ceres as a comparative planetary process

**DOI:** 10.1038/s41467-020-17184-7

**Published:** 2020-08-10

**Authors:** P. Schenk, J. Scully, D. Buczkowski, H. Sizemore, B. Schmidt, C. Pieters, A. Neesemann, D. O’Brien, S. Marchi, D. Williams, A. Nathues, M. De Sanctis, F. Tosi, C. T. Russell, J. Castillo-Rogez, C. Raymond

**Affiliations:** 1 Lunar and Planetary Institute/USRA, Houston, TX USA; 2 Jet Propulsion Laboratory/Caltech, Pasadena, CA USA; 3 Johns Hopkins University-Applied Physics Laboratory, Laurel, MD USA; 4 Planetary Science Institute, Tucson, AZ USA; 5 Georgia Institute of Technology, Atlanta, GA USA; 6 Brown University Providence, Providence, RI USA; 7 Freie Universitat Berlin, Berlin, Germany; 8 Southwest Research Institute, Boulder, CO USA; 9 School of Earth and Space Exploration, Arizona State University, Tempe, AZ USA; 10 Max Planck Institute for Solar System Research, Goettingen, Germany; 11 Istituto di Astrofisica e Planetologia Spaziali, INAF, Rome, Italy; 12 University of California, Los Angeles, CA USA

**Keywords:** Astronomy and planetary science, Asteroids, comets and Kuiper belt, Cryospheric science

## Abstract

Hydrothermal processes in impact environments on water-rich bodies such as Mars and Earth are relevant to the origins of life. Dawn mapping of dwarf planet (1) Ceres has identified similar deposits within Occator crater. Here we show using Dawn high-resolution stereo imaging and topography that Ceres’ unique composition has resulted in widespread mantling by solidified water- and salt-rich mud-like impact melts with scattered endogenic pits, troughs, and bright mounds indicative of outgassing of volatiles and periglacial-style activity during solidification. These features are distinct from and less extensive than on Mars, indicating that Occator melts may be less gas-rich or volatiles partially inhibited from reaching the surface. Bright salts at Vinalia Faculae form thin surficial precipitates sourced from hydrothermal brine effusion at many individual sites, coalescing in several larger centers, but their ages are statistically indistinguishable from floor materials, allowing for but not requiring migration of brines from deep crustal source(s).

## Abstract (web summary)

Dawn mission’s second extended phase provided high resolution observations of Occator crater of the dwarf planet Ceres. Here, the authors show stereo imaging and topographic maps of this crater revealing the influence of crustal composition on impact related melt and hydrothermal processes, and compare features to those on Mars, Earth and the Moon.

## Introduction

**Fig. 1:**
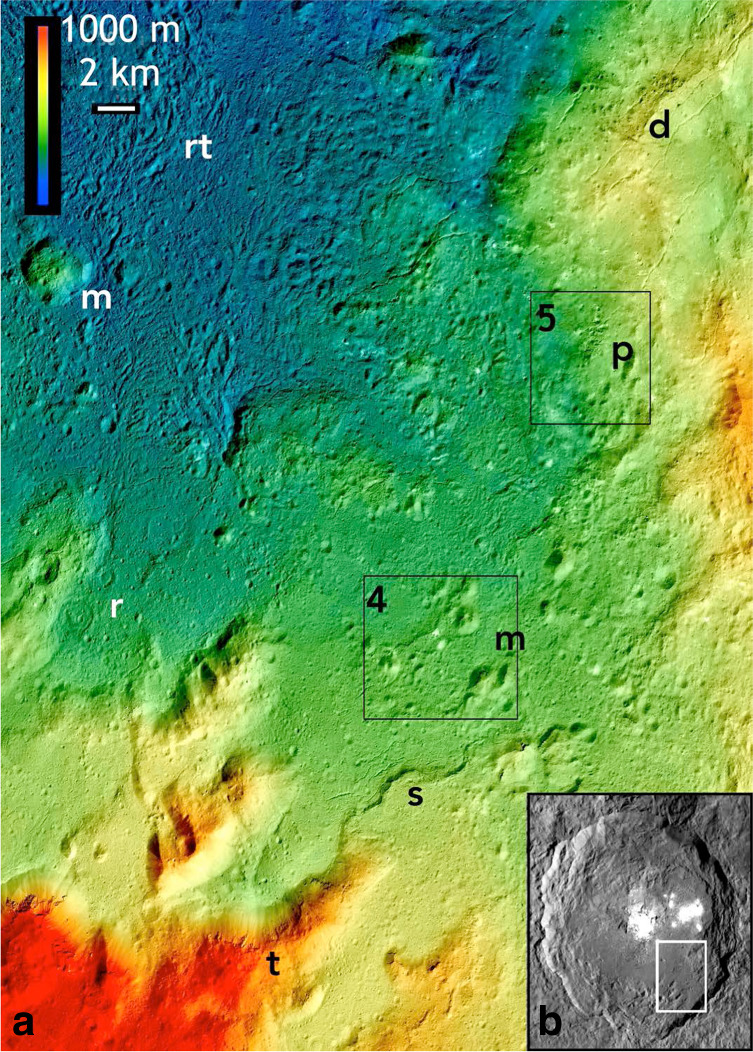
Portion of Dawn XM2 mosaic of southeastern floor of Occator. Color coding shows topographic relief including planar lobate floor deposits (LFD) over most of the scene that are ponded at higher elevation within the terrace zone at bottom. Squares show locations of Figs. 4 and 5. Letters indicate locations of several major features described in text: (t) terrace blocks partially buried by LFD, (s) inward-facing scarps 100–200 m high, (m) irregular floor mounds, (r) shallow ring depressions, (rt) ropey textured regions of LFD, (p) pit cluster, (d) field of dark knobs. Horizontal scale bar is 1000 m; vertical color scale bar shows 2 km of relief. Inset map of Occator (b) shows location of figure.

Hydrothermal fluids and deposits in terrestrial complex craters are important as potential habitats for thermophiles on early Earth^1^, and the Dawn discovery of hydrothermal deposition on the floor of the 92-km-wide central pit crater Occator^2^ and at limited locations elsewhere on the much smaller dwarf planet (1) Ceres^3^ represent an important new planetary body on which to examine these processes. Global imaging at ~35 m pixel scales revealed that Occator, the Tycho of the Asteroid Belt, is unique among craters >50 km on Ceres in that original formation morphologies are relatively intact relative to older degraded craters^4–6^. An extensive planar lobate floor deposit (LFD) in southeast Occator covers ~40% of the otherwise hummocky and ridged floor units^7–9^. LFD morphologies at these scales^9^ and modeling of impact heating^10^ suggested formation either as impact-derived deposits reminiscent of those observed in large craters on the Moon^9,11^ (e.g., Tycho, Jackson) and Earth (e.g., Manicougan^12,13^), or as post-impact volcanic outflows from deep subsurface water-rich reservoirs^7,14–16^, with important implications for Ceres’ internal thermal and geochemical structure. Similarly, bright salt-rich deposits (including Na-carbonates) in the central pit region at Cerealia Facula^4,6^ and within the LFD at Vinalia Faculae^2^ indicated that salt- and carbonate-rich fluids were exposed on the surface in a process involving either impact-derived or deep-rooted hydrothermal fluids, or both. The physical mechanisms by which any of these materials were deposited were not resolved at these scales, however. Key objectives of Dawn high-resolution observations across Occator^17^ (Fig. 1) in its second extended mission (XM2) were to ascertain emplacement mechanisms of floor deposits and the role of volatiles in their formation.

**Fig. 2:**
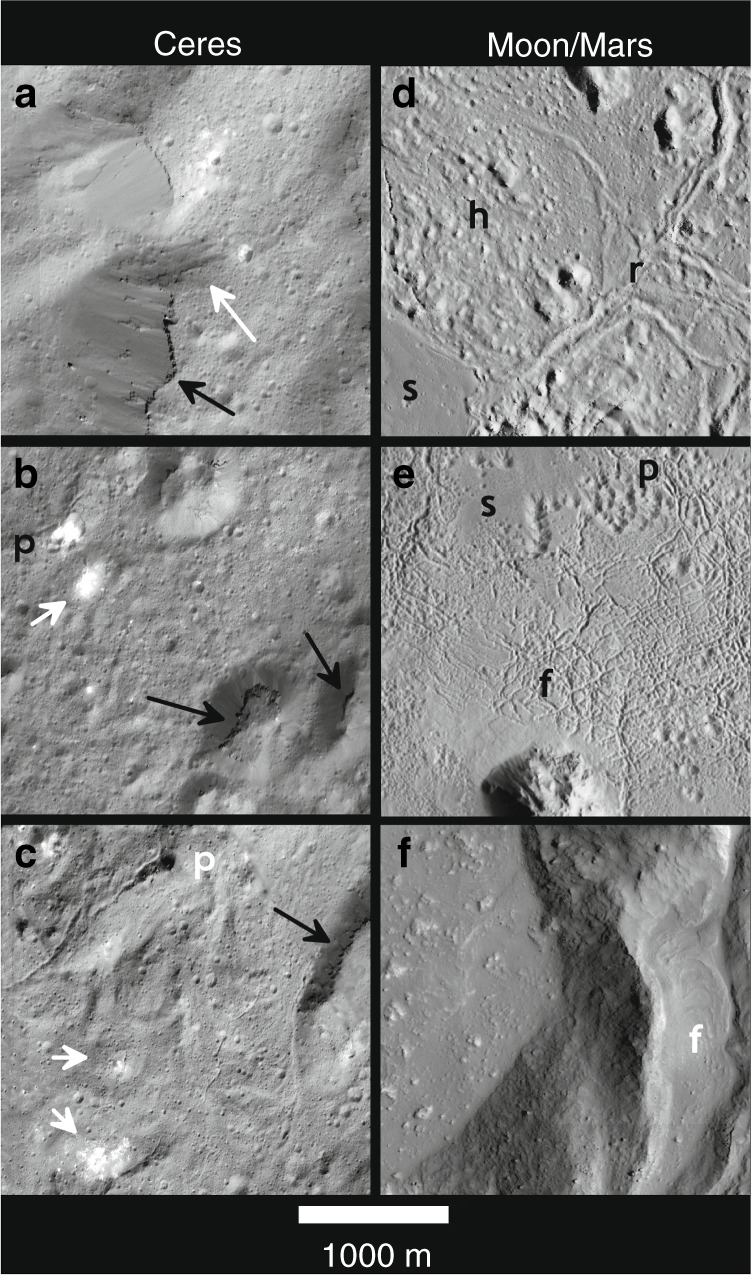
Floor textures at Occator and large lunar and martian craters. Ceres **a**–**c**: **a** Mantling deposits of impact melt, forming scarps on the crests and flanks of terrace units, with arrows pointing to topographic gap and striations of flow downslope from right to left and scarp-forming unit on terrace block; **b** bright floored oval depression, p, and bright conical mound (white arrow) in southeastern Occator floor of possible pingo-like origin. Mounds at lower right are capped by a resistant unit (black arrows). This site is shown in greater detail in Fig. 4a; **c** lobate ridged flow units within main lobate floor deposit, with elongate pit, p, transitioning to elongate fissure, arrows pointing to low bright deposits, and black arrow to scarp-forming unit on terrace block. Moon and Mars **d**–**f**: **d** variety of floor morphologies typical to lunar floor fill deposits: smooth, s; hummocky, h; and ridged r textured materials interspersed with knobby mounds in Jackson crater on the Moon (*D* = 71 km); **e** Smooth, s; pitted, p; and fractured, f, floor materials in southern Tooting crater on Mars (*D* = 28 km); **f** Smooth floor material with angular mounds in martian crater Pangboche crater (*D* = 10 km). Lobate ridged flow, f, similar to those on Occator **c** are perched on elevated terrace block. Ceres images were acquired at pixel scales of 3.5–8 meters, Moon and Mars images at ~1–5 m. North is up in all Ceres views; Moon/Mars views have been rotated with North down to present all views with the Sun to the right of the viewer.

**Fig. 3:**
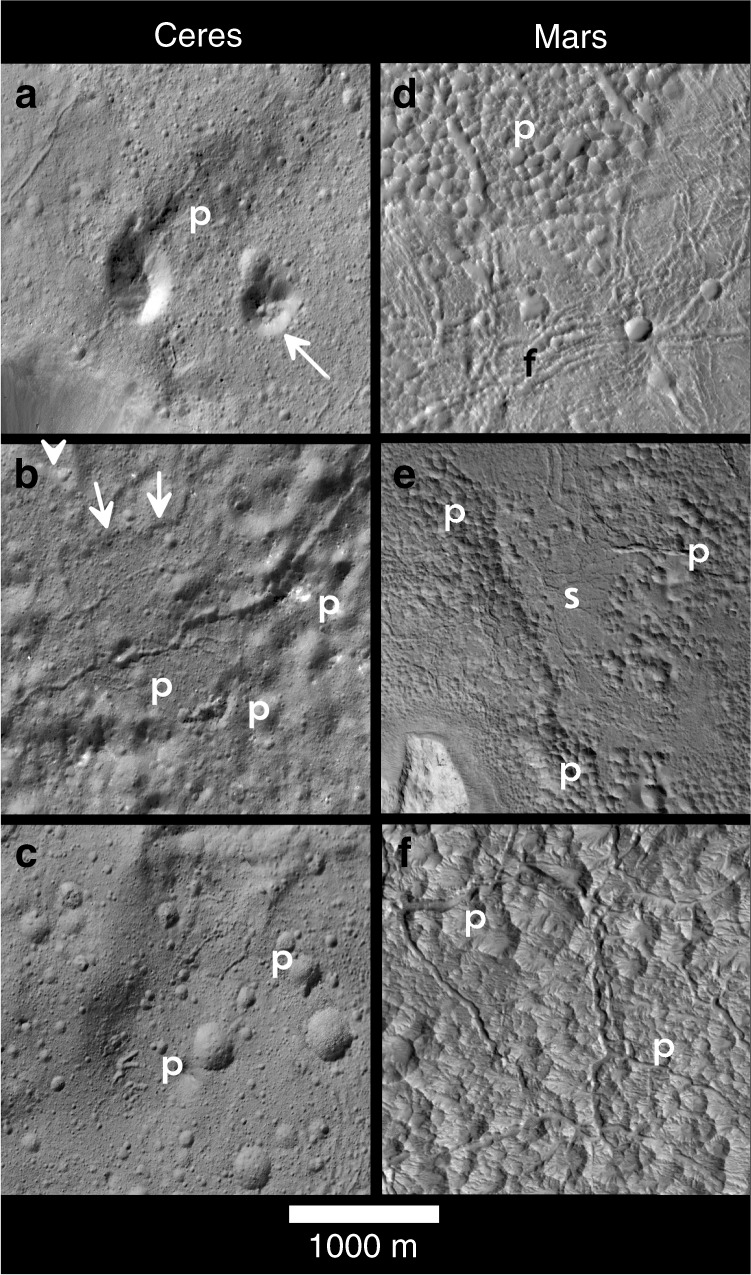
Non-impact pits and depressions on the floor of Occator and Martian craters. Ceres **a**–**c**: **a** elongate irregular-shaped dark pit, p, at base of floor mound in south Occator crater. Arrow indicates pit crested floor mound of possible pingo-like origins though pits could just as well be later impact craters; **b** sinuous troughs in association with irregular non-impact depressions, p, in LFD in southeastern Occator, with low scarps (arrows), indicating local flow fronts within the LFD; **c** irregular-shaped pits, p, and sinuous fissures within LFD materials north of the central pit of Occator. Mars **d**–**f**: **d** fractured, f, and pitted floor materials, p, in unnamed martian 51-km-wide crater (10°N 94°E); **e** smooth, s, and pitted floor materials, p, in northern Tooting crater on Mars; **f** pitted and fractured, f, floor material in Mojave crater (*D* = 60 km) on Mars. Ceres images were acquired at pixel scales of 3.5–8 meters, Moon and Mars images at ~1–5 m. North is up in all Ceres views; Moon/Mars views have been rotated with North down to present all views with the Sun to the right of the viewer.

Here, we test using XM2 stereo and DEM data whether water migration and freezing led to localized frost heave and surface uplift in a manner analogous to pingos^18^, whether volatile release owing to crystallization of melts occurred, and whether carbonate deposition occurred by localized brine effusion, fountaining and ballistic emplacement, and/or deposition in transient lacustrine settings^14–16^, or by another mechanism. We also compare the preserved impact and hydrothermal-related deposits at Occator formed in the salty and icy (and hence wetter) outer layers of Ceres^2,19–23^, with those on the mostly anhydrous basaltic and anorthositic lunar crust, the ice-enriched basaltic crust of Mars^24–27^, and with the more eroded hydrothermal deposits in the water-enriched silicic crust of Earth. Combined Dawn observations indicate a mixed crustal composition for the dwarf planet Ceres. The low density and high strength crust inferred from crater morphologies and topography relaxation has been interpreted as a mixture of ice (up to 40 vol.%), hydrated salts and silicates (<20 vol.%), and clathrate hydrates^21,22^. Available constraints indicate that the martian crust is dominantly anhydrous basaltic with ~5–15 wt% water in the near-surface regolith and a few percent water in the form of hydrated minerals and water ice down to at least 10 km depth^24–27^, although concentrations could be much higher locally. Despite dust mantling and recent gully formation on steep slopes in even young craters^28^, original textures can be discerned on recent well-preserved Mars craters (Figs. 2 and 3), the largest and most analogous to Occator being 60-km-wide Mojave^28^. On Earth, detailed field mapping is required to characterize hydrothermal deposition owing to pervasive erosion of larger complex craters, but provides critical constraints on subsurface hydrothermal activity^1,29^. If Europa Clipper or the ESA Ganymede mission confirm that Callisto’s outer layers are a partially differentiated^30,31^ mixture of ice and hydrated silicates^30,32^ then this body might also be a compositional analog to Ceres. Unfortunately, imaging resolution of well-preserved crater morphologies on Callisto is no better than ~125 m/pixel, but observations at Occator could be relevant when new Callisto data are returned.

## Results

### Rim features and mantling impact melts

Although many rim features at Occator are common to lunar craters, including boulders fractured in place and outcrops of coherent units^33^, overhangs of coherent rock layers and widely scattered meter-scale bright outcrops of possible carbonates near the rim crest are distinct to Occator (Supplementary Figs. 1–5). Ponding of solidified melt materials in closed depressions at different elevations within terrace and floor units (Fig. 1, Supplementary Figs. 6–8), a morphology common to impact melt deposits on the Moon and Mars, was also well documented at Occator^9^. Low-relief mantling units and downslope striations (Fig. 2a, Supplementary Figs. 3–5) indicative of flow in topographic gaps are now observed, consistent with draping, downslope flow, and local ponding of melt deposits broadly over most of the crater floor, and confirming that these are impact-generated deposits. These mantling units often form cliffs up to 10 m high where they encounter steep slopes, indicating that these units formed a coherent and resistant surface layer of comparable thickness.

Impact-generated melt deposits on Ceres are likely to be very similar in composition to the bulk composition of its outer layers^18–22^: melted ice mixed with unmelted salts and phyllosilicates, with both fine and coarse fragments and materials suspended and dissolved in aqueous solution and forming an impact melt that is mud-like in composition and consistency. Thus, impact melts on Ceres are likely much wetter than on terrestrial planets. These muddy impact brine-melt slurries on Ceres will be mobile while molten and behave much like (instantly emplaced) volcanic deposits, as recently demonstrated experimentally in a Mars context^34^.

Lobate flow features several kilometers wide and with relief of 10’s to ~350 m (Fig. 2c, Supplementary Figs. 7 and 8) are also common across Occator^9^. Low arcuate ridges are now resolved on most of these flow lobes, confirming widespread lateral flow of mobile material on the crater floor^6,9^, particularly in the northeast and northwest quadrants. A few flow lobes are observed merging into broader flow units at lower elevation or overlapping with other flow lobes, indicating overlapping deposition (or outbreak) flow events as these larger units were emplaced. These types of thicker large-scale flows lobes are significantly more common within Occator than on the terrestrial planets, where impact melt flows have very low relief and smaller dimensions^35^. This difference in morphology suggests that mud-like impact melts at Occator could have had effective viscosities an order of magnitude higher than melted basalt or anorthosite, depending on compositions and emplacement conditions that are poorly constrained at present.

The extensive distribution of mantling and flow materials across Occator documented in XM2 data (Figs. 2 and 3, Supplementary Figs. 4 and 5) is consistent with rapid emplacement and mobilization of large volumes of melted material during the impact event. Burial of terrace units in the southeast sector and lobate flow margins up to ~400 m thick along the northern margin of the LFD indicate thicknesses of >500 m and ~200 km^3^ of melted material (a volume that cannot be accounted for by a putative collapsed central peak at the central pit of Occator^9^). Mantling materials observed elsewhere are much thinner, ~10 m or less (Fig. 2, Supplementary Figs. 4 and 5). In the southeast sector (Fig. 1), floor mounds and inward-facing sinuous scarps 100–200 m high are often capped by cliff-forming resistant layers (Figs. 1, 2b, and 3) similar to those seen in the mantling units on terraces, suggesting rapid cooling and solidification of the outer layer of mobile impact deposits that were stranded on high terrains during or after emplacement. This mud-like, brine-melt impact slurry coated large areas, ponded in closed depressions and flowed downslope to form lobate lava-like flows^34^ (Supplementary Figs. 7 and 8) but also quickly formed a flash-frozen upper carapace and the resistant cliffs (Figs. 1–3), features not generally observed on lunar or martian craters.

Subsequent movement from south to north of the still molten interior of the southeastern LFD toward the mostly unfilled depressed northern crater floor^6,9^ may have formed the lobate flow features along the northern LFD margin (Fig. 2). Withdrawal to the north would require subsidence of the southeastern LFD and could explain the inward-facing cliffs that parallel the southeast crater rim and terrace scarps (where subsurface buried terrace scarps would impede lateral flow) and the resistant cliff-forming units stranded on the tops of many mounds (which were originally buried under the deposit) (Figs. 2 and 3).

### Endogenic mounds

An unresolved question from orbital mapping data was whether the irregular-shaped floor mounds are impact debris or are related to local upward deformation of the surface owing to subsurface intrusion and freezing of ice, a process related to pingo formation^18^ on Earth. The numerous mounds observed in XM2 stereo images form mesa-like to conical massifs ≤1750 m wide and ≤300 m high and of various shapes scattered across the southern LFD. These mounds have similar albedos and surface textures as surrounding floor materials, indicating they are of comparable compositions and ages. Summit fractures or vents of the type observed on terrestrial pingos^18^ and related to periglacial-style water intrusion and uplift and fracturing are not preserved on most of these mounds, although a few mounds do feature cryptic summit structures (which could also be post-formation impact craters). Most irregular-shaped neutral-colored floor mounds thus resemble the floor mounds observed on fresh larger craters on the Moon^11^ (where ice does not occur in significant quantities) and Mars (Figs. 2 and 3) and are more likely large fragmental impact debris piles abutted by rapidly emplaced low-viscosity LFD materials.

A subset of smaller rounded conical knobs <300 m across on Occator (very ~5% of all mounds) (Figs. 2 and 3, Supplementary Fig. 9) are bright relative to other crater material and can have a more yellowish color relative to other bright materials on the floor. These mounds could be small impact debris piles composed in part of carbonate-rich material, but their conical shapes and unusual coloration indicate they are the best and only likely candidates for post floor emplacement periglacial-style^36^ surface uplift by intrusion or expansion of carbonate-rich material from below. Some of these bright mounds also feature cryptic pitting or surface disruption that could be pingo-style summit fracturing^18^, but it is difficult to distinguish these small features from erosional effects or post-formation small cratering.

### Endogenic pits and troughs

Exhalation and release of volatiles driven out of solution from the crater floor and melt deposits as impact heating subsided^10^ was predicted owing to the high volatile content of Ceres outer layers^19–22^ and derived impact deposits. Large arcuate troughs crossing Occator^4,37^ are now resolved as sharply defined V-shaped troughs that crosscut all features and pinch-and swell from near 0 to ~250–350 m width over similar distances, forming deep elongate oval pits in some areas. Dawn XM2 imaging resolves initial unmodified narrow troughs ~10–20 m wide connecting the sharp-rimmed wider trough segments (Supplementary Fig. 10), demonstrating that the troughs begin as narrow fractures with little dilation that are later widened by non-uniform mass wasting. The large volumes of these structures point to significant volume loss. In many cases the bottoms of the troughs display a single row of very small pits <10 m wide (Supplementary Fig. 10), consistent with drainage of regolith into subsurface fracture voids under modest dilation, or venting of volatiles from the deeper fractures.

Abnormally shallow ring-shaped depressions up to ~1 km across (Fig. 1, Supplementary Fig. 11) common in the southern half of the crater^38^ are resolved down to ~10 m sizes and are (with a few exceptions) no more than a few 10’s of meters deep. The formation of secondary craters back onto Occator itself has been demonstrated numerically^39^ and documented in crater counts^40^ (compromising small crater statistics). From this, we argue that many of these shallow ring depressions are secondary craters that formed rapidly, i.e., before thicker LFD materials could completely crystalize into solidified formations and thereby contaminating crater statistics. The shallow ring morphologies of these unusual craters are also consistent with impact experiments into viscous clay-water slurries^41^.

The inferred age of Occator^40^ is a few to as much as ~20 myr. Even so, all floor units are extensively pitted with small roughly circular depressions <500 m across (Figs. 2 and 3) that could be impact or subsurface volatile release pits^42^ (which would imply much younger inferred ages and very extensive endogenic activity). Most of these depressions are broadly circular in shape, ubiquitously distributed and follow steep power-law size distributions and are inferred to be post-impact general cratering, though we admit that circular explosive pits could be difficult to distinguish from regular impact cratering at these scales.

**Fig. 4:**
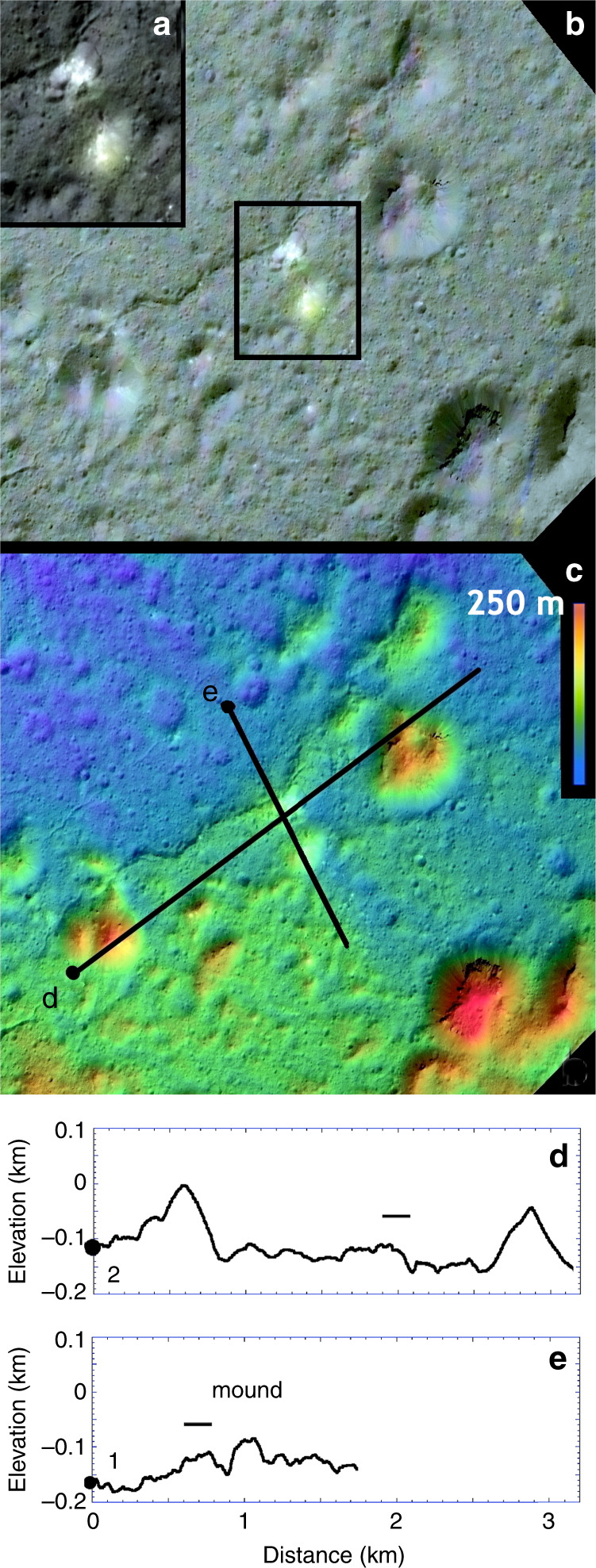
Ovoid feature and bright mound on lobate floor deposits of Occator crater. XM2 mosaic **a**, **b**, XM2 stereo-derived DTM (**c**; shown as color coding of mosaic; color scale shows 250 m of relief), and topographic profiles **d**, **e** across lines shown in central panel. Ovoid feature is 0.3 by 0.15 km-wide ovoid structure adjacent to sinuous scarp in LFD (16.9°N, 242.0°E), and horizontal bar in profiles. Inset **a** shows contrast-enhanced view of multiple bright spots within ovoid structure as well as adjacent bright mound with relatively yellowish color. Ovoid structure lies on a very low rise and may have been uplifted. Conical and knobby mounds flank the scarp to either side. **a** and **b** are color composites generated by assigning 965, 749, and 555 FC filter images to RGB channels, respectively.

**Fig. 5:**
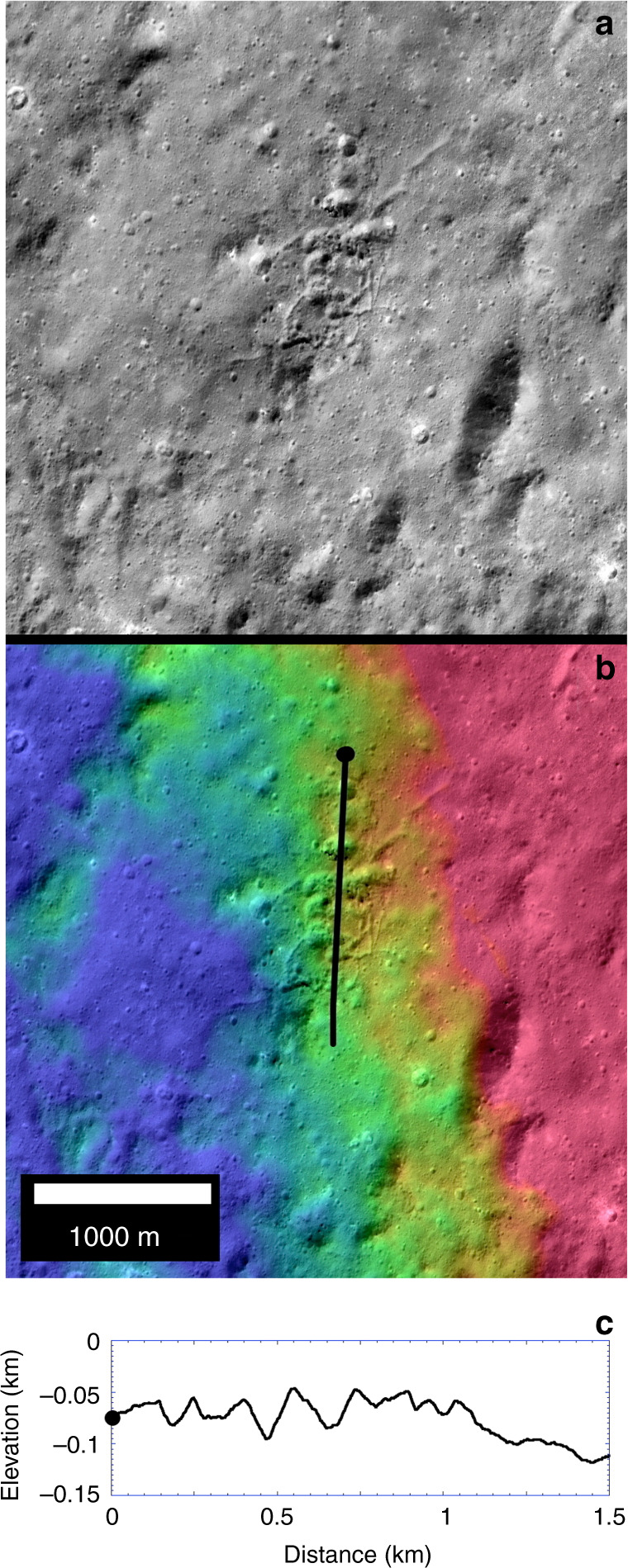
Cluster of ovoid pits on rugged floor unit of Occator crater. XM2 mosaic (top), XM2 stereo-derived DTM (center; shown as color coding of mosaic; color scale shows 250 m of relief), and topographic profile (bottom) across line shown in central panel. Mosaic and topography of clustered pits in hummocky and ridged floor material adjacent to LFD in southeastern Occator. These pits are unusually deep for their size and are associated with interconnected sinuous troughs and hence not secondary craters. Elevation extremes are saturated in this view to highlight relief of pits.

Limited occurrences of definitive irregularly shaped non-impact endogenic pitting are now resolved across Occator (Figs. 1–4). These take the form of ovoid to kidney-shaped depressions <250 m wide in widely scattered locations on both the LFD and hummocky floor deposits (Figs. 3–5, Supplementary Figs. 9, 12, and 13). These form either singly or in clusters (Figs. 3–5), are deep or very shallow, and are distinct from the shallow circular rings and background cratering described above by virtue of their noncircular shapes, proximity to sinuous troughs on the LFD described next, or geologic association with a web-like network of fractures (suggesting localized uplift or surface disruption (e.g., Fig. 5)). Other examples are distinguished by brighter spots or deposits on their floors (Fig. 4). These distinctly noncircular features are likely formed by volatile release or outflow late in the cooling of the crater floor and floor deposits as residual impact heat decayed following the main impact heating event^10^.

The formation of flow channels by water-rich fluids or slurries on the crater floor during outgassing of liquid water onto the surface was also considered before XM2. The XM2 images reveal numerous smaller-scale curvilinear and sinuous troughs typically ≤100 m wide and up to 7 km long (Fig. 3, Supplementary Fig. 14) that are very different in scale and morphology from the large-scale linear pinch-and-swell troughs that cross the crater floor. Some of the sinuous troughs must be fractures as stereo reveals they cross over prominent topographic rises with different slope gradients or cut adjoining units (Supplementary Fig. 14). These are possibly related to contraction or uplift of the LFD deposit and crater floor during post-impact cooling and solidification^4^. Other sinuous troughs lie within the planar LFD units (Fig. 3) and could be associated with the emplacement process by either cooling contraction, fracturing of an outer layer during lateral flow, or to surface fluid flow and erosion. In several cases, these sinuous troughs have branching patterns, are spatially associated with one or more of these endogenic pits described above, and are flanked by low scarp-bounded layers (Fig. 3, Supplementary Fig. 6), possibly implicating subsurface volatile release and outflow of minor amounts of post-emplacement melts through surface vents and fissures.

The scattered smaller irregular-shaped endogenic pits, domes, and narrow sinuous troughs within Occator (Figs. 2–5, Supplementary Figs. 9–14) are rare in lunar or martian craters. The endogenic pits on the floor of Occator are distinct from the much larger and more abundant pits within melt deposits of larger well-preserved martian^43^ and vestan^44^ craters (Fig. 3). The martian and vestan pits are abundant and usually closely spaced (Fig. 3), forming adjoining walls in a honeycomb style and are attributed to explosive vents from volatile release^43–46^. Though some are misclassified secondary craters, large-scale pits broadly similar to the martian examples occur in the large cerean craters Ikapati and Haulani^47^, and possibly elsewhere. The lack of pitting of this type at Occator and several other large craters suggests compositional heterogeneity in the Cerean crust or differences in impact conditions such as velocity.

The endogenic pits and pit clusters, non-tectonic sinuous troughs and perhaps the bright knobs within the LFD and adjacent crater floor materials (Figs. 2–5; Supplementary Figs. 6 and 9–14) are consistent with continued release of volatiles from beneath the crater floor and LFD after emplacement. Crystallization of the impact melt slurry at Occator likely involved sediments coming out of suspension and salts and ice coming out of solution, creating secondary fluids and gases that may have extruded onto LFD surfaces^15,16,36^ to form some of the local pitting and perhaps some of the sinuous troughs and brighter knobs. Cooling and crystallization of impact-heated materials below the crater floor may also have driven volatiles to the surface^10,14–16^.

Despite the large extent, thickness, and inferred high volatile content of these floor fill deposits, neither endogenic mounds nor pits are dominant landforms on the floor of Occator. This comparative difference to the much larger and densely spaced pits on the floors of large martian craters (Fig. 3) suggests that their formation may have been inhibited on Occator and perhaps on Ceres generally relative to those on Mars^43–46^. These differences could be related to the abundance of phyllosilicates in the outer layers of Ceres^19,20^. We speculate that the extensive cliff-forming layers 10’s of meters high on the upper surfaces of mantling impact melt flow units across the LFD and terraces of Occator (Figs. 1–3, Supplementary Figs. 4–6) may be clay-rich layers that formed over the cooling and solidifying impact melts and crater subsurface. These resistant mud-like layers may have had relatively low permeability and could have inhibited volatiles from access to the surface relative to Mars, except where locally breached. As surface units exposed to space they likely crystallized rapidly during the flash freezing of the upper surfaces of the pervasive melt. The limited expression of volatile release features across the volatile-rich floor of Occator could be owing to (1) only small amounts of volatiles driven out of solution and onto the surface during solidification of the melt deposits and cooling of the interior, (2) volatiles remaining where they accumulated within the impact melt deposits, and/or (3) surface layers preventing them from accessing the surface.

### Hydrothermal carbonate deposits at Vinalia Faculae

**Fig. 6:**
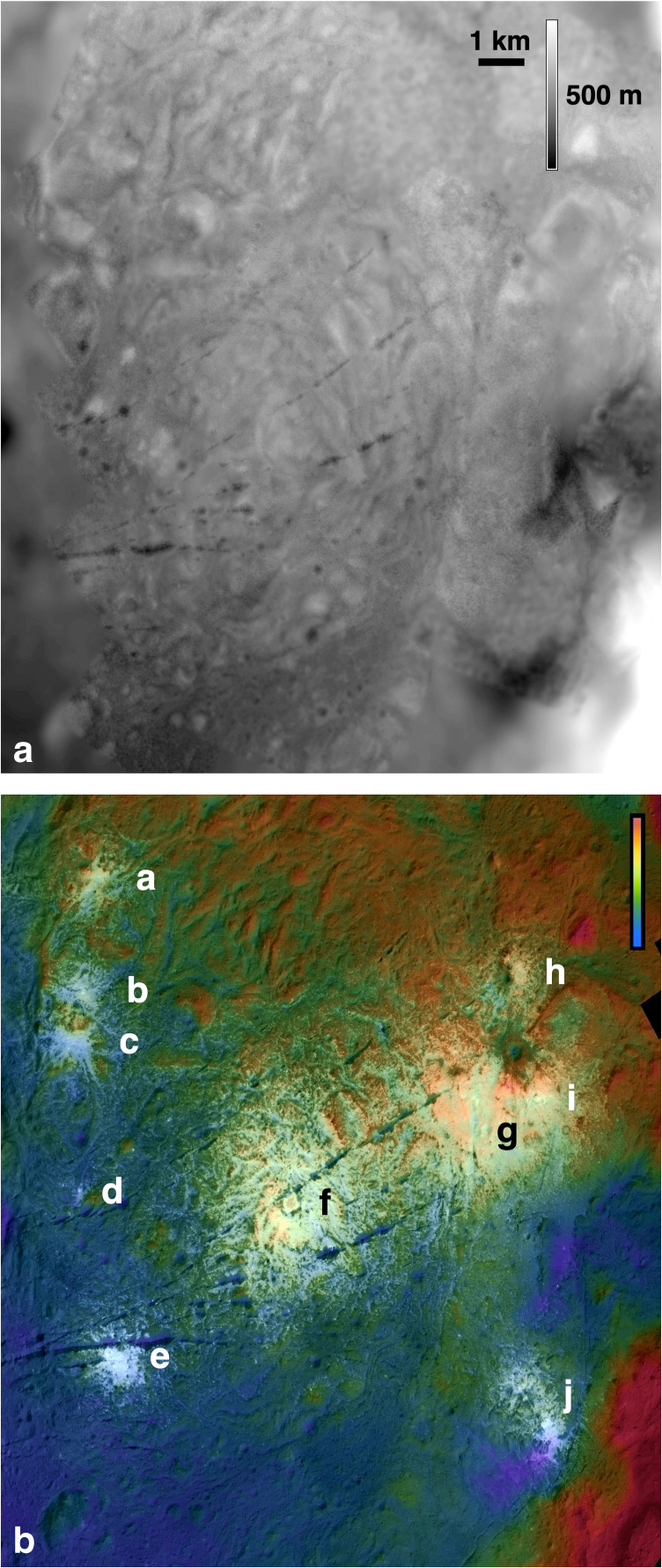
Topography and morphology of bright carbonate deposits in Vinalia Faculae. **a** High-resolution DTM of northern LFDs derived from Dawn XM2 stereo imaging. Elevations shown in grey (dark = low). **b** Same elevation data shown color-coded for elevation (blue = low, red=high) combined with XM2 high-resolution mosaic of same area, scaled to −250 to +250 m. The major carbonate deposit concentrations or bright centers are labeled **a**–**j**.

The 10 or so distinct concentrations or centers of bright deposits at Vinalia Faculae^4,48^ on the ridged eastern LFD (Fig. 6, Supplementary Figs. 10 and 14–18) are the most obvious and laterally extensive manifestation of volatile redistribution within the Occator floor deposits, namely hydrothermal redeposition of salts and carbonates^2,3^. Despite well-documented subsurface exposures of hydrothermal fractures and conduits on eroded terrestrial craters^1,29^, these deposits have no direct surface analogs on terrestrial planets, likely owing to the surface degradation processes prevalent on Mars and the Earth. The surface emplacement mechanisms of carbonates are related to the subsurface reservoirs and conduits and provide a key test of how hydrothermal systems work on extraterrestrial bodies^1,29^. Hypotheses include (1) brine seepage through innumerable small (meter-scale) vents^9^, (2) energetic fountaining and ballistic emplacement^3^, and/or (3) precipitation and sedimentation of mineral deposits in static if transient lacustrine environments. Duration of emplacement is also important as durations of ~10^7^ yr or more would require deep reservoirs^14,17^, but shorter periods would imply shallow reservoirs such as the LFD itself, owing to the limited timescales over which residual impact heat would remain^10,15–17,49^.

**Fig. 7:**
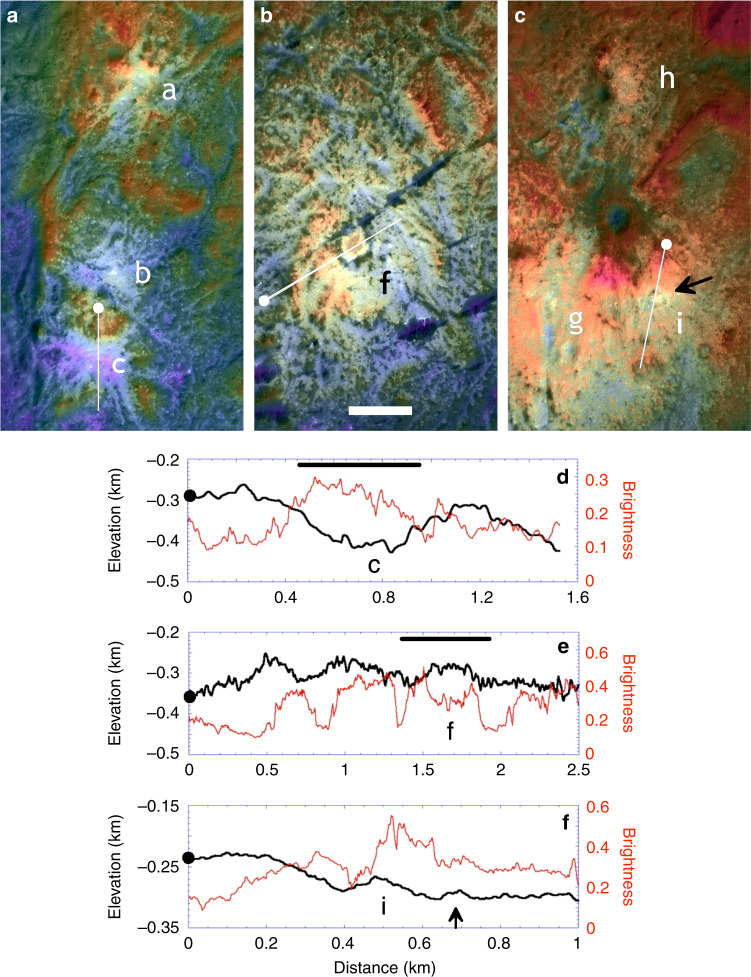
Topography of major bright patches at Vinalia Faculae. **a**–**c** Colorized digital mosaics of major bright patches, color-coded for elevation (blues are low, reds are high; see Fig. 6 for color scale). Interior labels refer to deposit bright centers described in text. Bright centers labeled **a**–**c** are controlled by local topography; center **f** features dark ring, center **g** is the most extended and contiguous deposit, and center **i** marks location of putative constructional dome at 20.36° N, 243.0° E (arrow). See also Supplementary Fig. 3 for stereo views, including dome **i**. **d**–**f** Topographic (black lines) and brightness (red lines) profiles across several bright centers within Vinalia Faculae. Profile **d** highlights correlation of low topography with bright deposits at center **c** in a typical western bright center. Profile **e** highlights dark ring and associated low topography at center of **f**. Horizontal bar in profile **c** indicates lateral extent of bright depression, and in **e** denotes extent of dark ring. Arrow in profile **f** indicates location of small dome in bright center **i**.

**Fig. 8:**
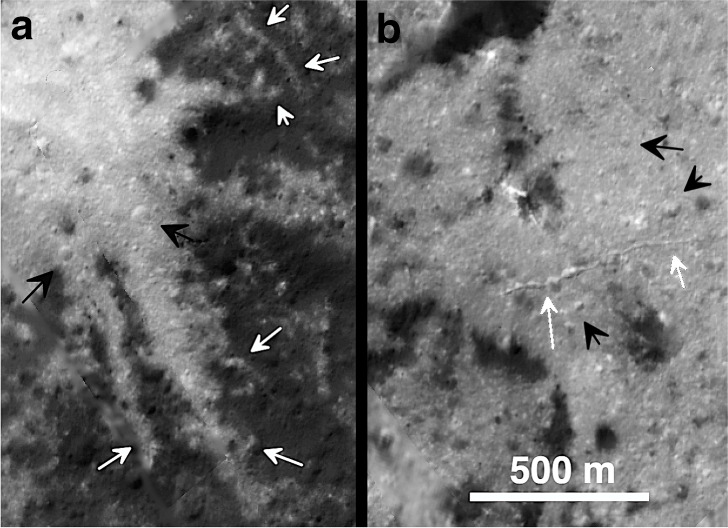
Surface textures within bright Vinalia Faculae deposits. Bright arrows in **a** indicate narrow ribbon-like deposits filling local depressions or crossing ridges within Vinalia Faculae bright center **c** in Fig. 6b (see also Supplementary Fig. 16 for stereo images) and narrow sinuous trough and pits in **b** that were active after bright deposits were emplaced within bright center **g** in Fig. 6b). Dark arrows highlight some of the largest of the many small craters on the surface of the bright carbonates. Areas are enlarged from Fig. 6b, without color coding.

Vinalia Faculae bright deposits have complex topographic relationships (Figs. 6 and 7; Supplementary Figs. 10 and 15–18) with the LFDs of eastern Occator crater floor on which they formed. These northern LFD consist of closely spaced elongate and triangular ridges of 50–125 m amplitude that form closed irregular depressions between them (Figs. 1 and 6, Supplementary Figs. 15–17). The major bright centers of Vinalia Faculae (a–j in Fig. 6) are resolved into bright central cores grading outward into progressively smaller discrete amoeboid or ribbon-like patches a few 10’s of meters across of decreasing brightness. The smaller patches often fill local depressions in the LFD but also cross the tops or flanks of topographic ridges or follow valleys upslope (Figs. 6–8). Thousands of smaller circular bright spots <10 m across are dispersed on the surfaces of these larger patches and the LFD itself.

The brightest western and southern bright centers form radiating lobate or amoeboid patterns ~1 km across that are strongly controlled by the local topography (centers (a–d) in Figs. 6 and 7; Supplementary Figs. 17 and 18). These deposits line but do not topographically fill several larger inter-ridge LFD depressions 0.25 to ~1 km across (centers (a–d) and (h) and (j) in Figs. 6 and 7). The similarity in depth to neighboring unlined depressions indicates deposit thicknesses of no more than a few meters, consistent with estimates based on measurements of dark-floored craters^48^. In central Vinalia Faculae, the two largest bright centers are resolved as broader contiguous deposits 2–3 km across (center (e) in Figs. 6 and 7, Supplementary Fig. 15), which cover topographic highs and lows. These larger spots also have undulating relief indicating mantling of LFD topography, grading into the amoeboid patterns described above. This overall pattern (Fig. 6) suggests that the peripheral bright centers (a–e) and (h and j) were less vigorous than the larger (f) and (g) bright centers.

The radially outward decrease in brightness of these deposits suggests that active deposition at each bright center contracted inward with time, with the outermost deposits fading in brightness owing to exposure to space. The numerous circular spots smaller than ~10 m across indicate that the later stages of deposition were dominated by point sources. Although the cores of the western and southern bright centers are confined in closed topographic depressions (centers (a–d) in Figs. 6 and 7, Supplementary Fig. 16), the smaller patches in the outer peripheries of the major bright centers are located on various slopes or tops of ridges. In some cases, they occur on one side of a ridge but not the other. The complex and inconsistent topographic associations and lack of definitive large singular source vents or constructions at most bright centers at Vinalia Faculae (Figs. 6 and 7; Supplementary Figs. 10 and 15–18) is more consistent with carbonate deposition via surface seepage and effusion of brines from below^3,9,15^ at innumerable submeter vents within the LFD, and broadly consistent with occurrences of hydrothermal deposits on Earth^50^. Those surface sites with low seepage rates or volumes formed isolated small bright spots or downslope ribbons where flow persisted, those with higher rates or volumes resulted in more extended and coalesced carbonate surface formations as fluid flow and deposition spread laterally. The central regions could also be more contiguous if there were more vents in these regions.

Constructional features from hydrothermal activity (e.g., travertine terraces) are lacking or not resolved. The unusual dark ring in the middle of bright center (f) (Figs. 6 and 7) is a candidate larger scale vent. The dark ring is associated with shallow troughs a few 10 s of meters deep flanking a low-bright polygonal rise, none of which, however, are distinct topographically or morphologically from the many similar ridges and depressions in LFD (Fig. 6; Supplementary Fig. 17). The best and only other candidate constructional feature resolved at Vinalia Faculae is a small 25 m-high and 140 m-wide lobate dome in the center of a 340 m-wide depression in bright center (i) (Figs. 6 and 7; Supplementary Fig. 18), suggesting domical extrusion or accumulation of a small amount of new material into a shallow explosion or impact pit; any others have been destroyed.

Degassing of these fluids in the vacuum of space probably resulted in rapid precipitation of the suspended and dissolved carbonates and salts directly onto the surface. Fountaining and ballistic deposition of carbonates at individual spots at meterscales^3,14–16^ is not excluded by XM2 observations but there is little if any observational evidence that it occurred extensively. The distribution of small bright spots and surface coatings on floors of closed depressions, the flanks of some ridges and the tops of others, and the formation of meter-scale ribbon-like deposits is not consistent with directional ballistic sources (i.e., fountaining) greater than a few meters in scale. Although lacustrine sedimentation within closed depressions is also suggested by the formation of some deposits on the floors of inter-ridge LFD depressions (and indeed small local ponding of brines may have occurred for very short periods before boiling off), the formation of smaller spots on slopes and ridge tops (Fig. 7) and the broader ridge and trough covering eastern bright centers (Figs. 6 and 7) also suggests that a lacustrine environment was not required for carbonate formation in most cases.

These observations also suggest that the subsurface beneath the major Vinalia Faculae bright centers was more densely fractured and permeable, permitting fluids easier access to the surface than elsewhere in Occator. The interplay between laterally asymmetric subsurface hydraulic gradients in radial brine flow regimes and the rugged local topography coupled with highly localized and sporadic fountaining may account for the asymmetric slope distributions and complex topographic relationships of carbonates in many locations.

The ages and duration of carbonate deposition at Vinalia Faculae are difficult to constrain but the suggested 2 myr duration of emplacement^49^ is likely unsupported owing to the large uncertainties associated with obtaining reliable small crater counts in young surfaces^51^, especially in such small areas in high contrast terrains^52^. Small pits <60 m across densely cover the surfaces of the bright deposits (Fig. 8, Supplementary Figs. 19–21). Though some of the pits on the surfaces of the carbonate deposits could be endogenic pits related to the release of carbonate-bearing volatiles onto the surface, the vast majority are circular and likely of impact origin and similar in appearance to the innumerable craters on LFD, with only rare examples exhibiting noncircular shapes. Assuming most of these circular depressions are impact related, our crater densities in the LFD indicate a crater retention age of between 4 and 8 myr (Supplementary Fig. 21), which may also give a crater formation age as these are likely impact melt products^9,40^. Crater densities for the VF carbonate bright centers (c, g, and h) (Figs. 6 and 8, Supplementary Fig. 21) indicate ~4 myr ages. Both sets are broadly consistent with reported ages^49^, but, with formal uncertainties of at least 0.5 myr, the estimated ages on the VF deposits are statistically indistinguishable from those observed in nearby LFD deposits (Supplementary Fig. 21). Only crater densities at bright center (i), where the constructional dome in Fig. 6 is located, were significantly lower, suggesting ages of ~2 ± 0.2 myr for this specific bright center. The significance of even this difference is in doubt as carbonate deposits of <10 m thick formed by surface seepage will mantle craters larger than ~10 m (our counting limit) and the degree of crater erasure may be locally variable. Also, more of these craters may be endogenic than is assumed. Thus, crater counts in these deposits likely reflect a combination of craters formed prior to, during and after carbonate deposition and suggest a prolonged though indeterminant formation period for the carbonates at VF.

The XM2 images further elucidate the complex relationship between the long arcuate floor troughs^4^ and the carbonates. The carbonate deposits at Vinalia Faculae mantle the narrow (circa 20 m-wide) linear segments of these troughs, but are crosscut by the wider pinch-and-swell segments that have been locally enlarged to ≤300 m by mass wasting (Fig. 6, Supplementary Figs. 10 and 15). Exposures of bright deposits along some fracture walls further confirm that the carbonate deposits are surficial deposits no >3–5 m thick^48^. Carbonate deposits can be seen to move downslope by mass wasting down some fracture walls in the form of narrow ribbons (Supplementary Figs. 10 and 15). Despite this, we observe no evidence of carbonates having been extruded from within the fractures, and most carbonates are not associated with any resolvable structures (though the crater floor is likely densely fractured and these likely were major conduits for hydrothermal fluids within Occator).

Well-preserved younger short narrow sinuous troughs (Figs. 2c, 3b, and 8) crosscut the carbonate deposits in a few locations. Although some carbonates could also have been deposited on these troughs after they formed, their morphologic sharpness suggests that they formed after most of the deposits were in place. The inferred formation sequence at Vinalia Faculae is (1) emplacement of LFDs, (2a) narrow arcuate fracture formation across much of Occator, including the area of Vinalia Faculae, (2b) formation of carbonates by brine seepage (both fracturing and seepage may have been prolonged over an uncertain period and likely began immediately after (1)), (3) enlargement of arcuate fractures to form the pinch-and-swell troughs, and (4) late-stage minor sinuous trough formation (Figs. 6 and 8, Supplementary Figs. 10 and 15).

### Impact-driven volatile redistribution—planetary comparisons

Similarities and differences in morphologies and distributions of impact melt deposits and hydrothermal mineral redeposition on the Moon^11^, Earth^12,13^, Mars and Ceres (Figs. 2 and 3, Supplementary Figs. 22–26) are related to differences in composition and impact conditions. Ponding of impact-melt-related LFDs in closed depressions at different elevations is similar to that observed in lunar and martian complex craters and established that impact melts were widely distributed across Occator^9^ (Figs. 1–3, Supplementary Figs. 22–25). Morphologies of pristine impact deposits vary among and even within individual lunar complex craters^11^ (e.g., Jackson, Ohm, Lowell, Rutherford, and Tycho). These take the form of ridged (or ropey), and knobby/hummocky and smooth plains, each of which find analogs at Occator (Figs. 1–3, Supplementary Figs. 22–25). The brighter small knobs scattered across the floor of Occator are distinct to Ceres, however, and indicate post-emplacement hydrothermal activity or at least exposure of distinct carbonate-rich materials by localized uplift, and the best evidence for post-impact periglacial activity^38^ on the floor of Occator (Fig. 4, Supplementary Fig. 6).

We observe several different styles of volatile related deformation on the floor of Occator. The >100 m thick and multi-kilometer wide lobate flows and flow margins at Occator^9^ (Fig. 2, Supplementary Figs. 6 and 9–12) are not common within lunar or martian craters, and are likely related to the unique mud-like impact melt composition on Ceres forming a more viscous or debris-choked material. The thick margins of these larger flows may indicate rheologies analogous to terrestrial ice-rich rock glaciers^53^. Roughly 5–10% of fresh martian craters, such as Pangboche^54^ (Fig. 2f), display a lunar-like morphology, including ridged lobate flow textures similar to those at Occator (Fig. 2) but conspicuously absent the floor pitting and layered ejecta deposits indicative of volatiles^11^. These martian craters are normally at higher elevations where subsurface ice content is inferred to be lower^11^ and illustrate the potential role of composition in modulating crater morphologies.

The locally dense fracture networks observed in complex craters on the Moon^11^ and Mars^44^ (Fig. 3; Supplementary Fig. 22 and 25) find no direct analog in Occator. Volume expansion owing to water freezing at Ceres may mitigate against the cooling contraction fracturing observed in some lunar craters. Some of the narrow unconnected sinuous troughs peculiar to Occator’s LFD and floor units (Figs. 2 and 3, Supplementary Figs. 6 and 13) are likely related to fracturing owing to gravitational adjustment of the crater floor, but others are associated with small pits and thin secondary lava-like^34^ muddy flow units (Fig. 3b, Supplementary Fig. 6) and likely represent surface modification owing to volatile release and fluid mobilization associated with the large volumes of impact melt and may be peculiar to Occator.

Hydrothermal fluids and deposits in terrestrial complex craters are concentrated as vug and vein systems within and proximal to the central uplift (corresponding to the bright deposits at Cerealia Facula^9^), within crater floor materials (including melts), and along the faults defining the outer terrace blocks^1^. Mineralization is dependent on target lithologies and with depth below the surface^29^, and higher temperature precipitates occur at depth on planetary bodies, including probably Ceres. The Vinalia Faculae deposits (Figs. 6 and 7) are too distant and unlikely to be related to terrestrial style vein systems associated with the (buried) distal edges of the Occator central uplift. Why they occur only in the eastern (and possibly thickest) sector of the vast LFD melt deposit remains unclear but the XM2 data reveal that they are not directly derived from the older narrow arcuate troughs (Figs. 6 and 7) and there is no evidence for structural control for most of the deposits. Rather the innumerable small bright spots and patches (which coalesce at the major bright centers (a–j)) and the complex topographic relations to ridged LFD deposits (Fig. 7, Supplementary Fig. 8) indicate that the Vinalia Faculae carbonates are likely formed from brine seepage through innumerable small vents, possibly from beneath the LFD itself.

In contrast to well-mapped impact-initiated hydrothermal impact systems at large complex craters on Earth^1,29^, the absence of carbonate deposits or endogenic features along the bases of rim wall terraces or across most of the crater floor (Vinalia Faculae excepted) confirms that the zone of hydrothermal activity beneath Occator was much more centrally restricted, or rim wall sources were shut off early and any bright carbonate deposits there have faded. The concentration of the densest carbonate deposition into the dozen or so major bright centers at VF suggests that brine volumes may have been limited or originated deep in the crust^10,16,17^, leading to focused flow centers. The potential role of deeper hydrothermal crustal reservoirs^17,48^ rather than mobilization of fluids in rapidly cooling subsurface impact melt reservoirs relies in large part on the ages of these deposits. Unfortunately the uncertainties currently associated with age determinations from crater counting on the deposits as described above (Supplementary Fig. 21) are sufficiently large that the duration of carbonate formation relative to the terrains they formed on could be within the ~10^6^ yrs limits imposed by the cooling of impact melt reservoirs^10,15,16^ or as long as a few myr^49^. Whether other lines of evidence are sufficient to require a deep long-lasting reservoir^17,48^ requires further study.

## Discussion

The differences in surface morphology of impact deposits on Ceres compared with the Moon and Mars shown here must be related to the higher fraction of salts and water ice in Ceres’ outer layers^19–21^ and to different impact conditions on Ceres (including ~40°K cooler near-surface temperatures^10^, 5-to-15 times lower surface gravity, and factor ~2 lower mean impact velocities^10^), which result in lower heating rates for a given size projectile on Ceres^10^ relative to Mars or Earth. These lower impact heating levels preclude melting of silicates on Ceres, resulting in muddy deposits that behave as volcanic flows^9,34^, and may result in a more centrally concentrated distribution of hydrothermal fluids and salt/carbonate surface precipitation compared with those observed on impact-derived materials and structures on the Moon, Earth, and Mars^1,29^. The irregular pits and pit clusters, thin lava-like mud flows, and sinuous troughs observed within the thick LFDs and other floor materials at Occator are both less common and are different in expression to those within lunar and Mars craters (e.g., Figs. 2 and 3, Supplementary Figs. 24–26). The manifestation at Occator of smaller widely scattered endogenic pits rather than the numerous larger closely spaced pits on Mars^43,44^ and Vesta^46^ (Figs. 1–3, Supplementary Figs. 24–26) is curious considering the higher volatile content of Ceres crust and its impact melt products (up to 60% ice and clathrates^21,22^ vs. ≤10% ice for Mars^26,27^). The rapid formation of extensive cliff-forming (Figs. 2 and 3, Supplementary Figs. 4–6) as possibly low permeability surface layers during initial crystallization of the melt deposits may have partially inhibited access of volatile fluids to the surface relative to martian craters during the prolonged cooling and solidification phases. Alternatively, low-temperature crustal components may be more easily vaporized or more explosively released on Mars than on Ceres, or water may have remained sequestered more extensively on Ceres than on Mars (where perhaps <3 wt% is inferred to be in hydrated minerals except in polar areas^24–27^). If chondritic materials^55^, including hydrated silicates^32^, are abundant Callisto’s surface, the higher impact velocities may also result in hydrothermal fluid redistribution and formation of precipitates of salts comparable to Mars or Ceres within larger fresh central pit craters on large icy satellites as well.

Here, we show that impact-related deposits on the floor of the very young Occator crater on Ceres exhibit distinctive morphologies indicative of volatile release owing to the melting and refreezing of water ice during and after impact. These features include ovoid and sigmoidal pits and pit clusters, sinuous troughs, low bright mounds. Carbonate deposits at Vinalia Faculae are revealed to be only a few meters thick and controlled by local topography of the ridged floor materials they formed on. The complex relationship of these deposits with topography is consistent with hydrothermal brine effusion and carbonate precipitation at innumerable small vents, coalescing at several larger centers. These morphologies are substantially different from those observed in large martian craters, indicating that while hydrothermal processes previously identified on Mars and Earth are also prevalent on Ceres the composition of Ceres’ outer layers strongly controls how these processes operate on each planetary body.

## Methods

Dawn Framing Camera (FC) stereo imaging of most of Occator floor acquired during XM2 at 3.5–8 m pixel scales (Fig. 1, Supplementary Figs. 1–5) are used here to construct registered mosaics and digital terrain models of relief with factors ~10 improvement in quality from prime mission orbital mapping topography^56^. We also use the stereo images to determine the stratigraphic and topographic relationships of LFD and salt-carbonate deposits to the terrains they formed on and to test hypotheses for their formation^14–17^. Dawn FC image registration and construction of XM2 stereo mosaics, tied to Dawn FC LAMO mosaics, was performed using USGS-ISIS (version 3.5.2.0) FC calibration and control net bundle adjustment software (https://isis.astrogeology.usgs.gov). Digital Elevation Models (DEM) were produced from registered Dawn stereo images using stereogrammetry software designed in the MATLAB toolbox, identical to that used to produce DEMs for the surface of Pluto^57^. Crater counts were completed from the FC mosaics and interpreted using the asteroid-based crater chronology^58,59^. Cratering ages were derived using hard-rock cratering scaling law with a low value of strength, 1e6 dyne/cm^2^ (see ref. ^59^ for additional details about cratering scaling laws). The target and impactor density were 1.3 and 2.6 g/cm^3^, respectively, and assumed an average impact speed of 5.1 km/s^59^.

## Supplementary information

Supplementary Information

## Data Availability

The Dawn FC imaging data that support the findings of this study are available on the PDS Small Bodies Node website at https://sbn.psi.edu/pds/resource/dawn/dwncfcL1.html and at https://sbnarchive.psi.edu/pds3/dawn/fc/DWNC7FC2_1B. Dawn FC LAMO mosaics are available at https://sbnarchive.psi.edu/pds3/dawn/fc/DWNCLCFC2_2/. The preliminary DEMs and associated image mosaics derived for this work are archived at the USRA Science Data Repository at https://repository.hou.usra.edu/handle/20.500.11753/1564. Stereo images presented in the Supplementary Information are archived at the NASA Photojournal website under Ceres.
