# Supplementary material for: Impact heat driven volatile redistribution at Occator crater on Ceres as a comparative planetary process
Source: Nat Commun. 2020 Aug 10;11:3679. doi: 10.1038/s41467-020-17184-7 (PMC7417549; doi:10.1038/s41467-020-17184-7)
Supplement: Supplementary file 1 — Supplementary Information [file 41467_2020_17184_MOESM1_ESM.pdf]

Supplementary Information for:

Impact heat driven volatile redistribution at Occator crater on Ceres as a comparative planetary process

Schenk, P., et al., Nature Communications, 2020

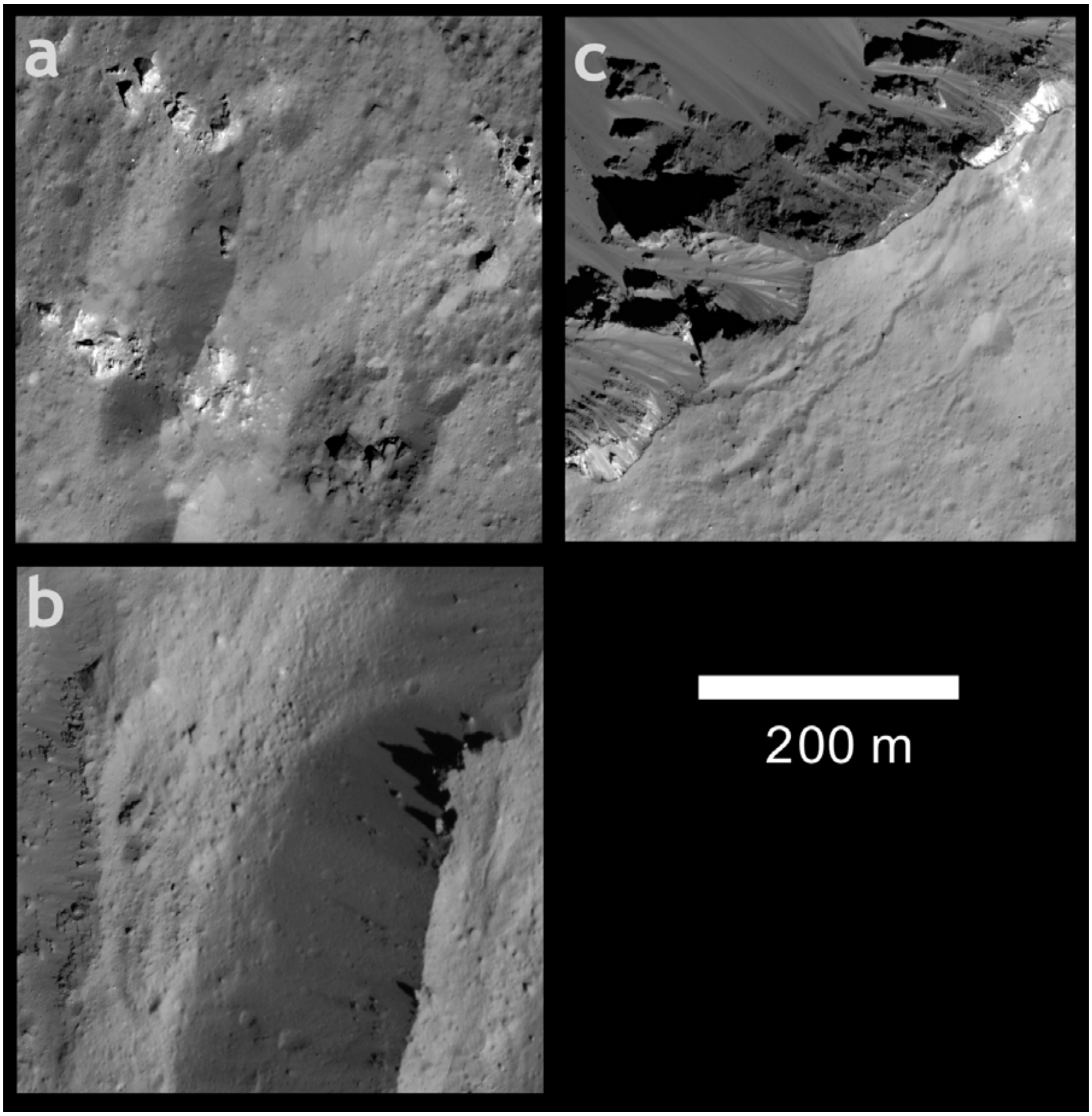

Supplementary Figure 1. Dawn XM2 images of Occator crater rim. Features associated with the rim of Occator crater include bright and dark boulders (a), overhangs of resistant units (b), and exposure rock layers and bright outcrops along rim crest (c).

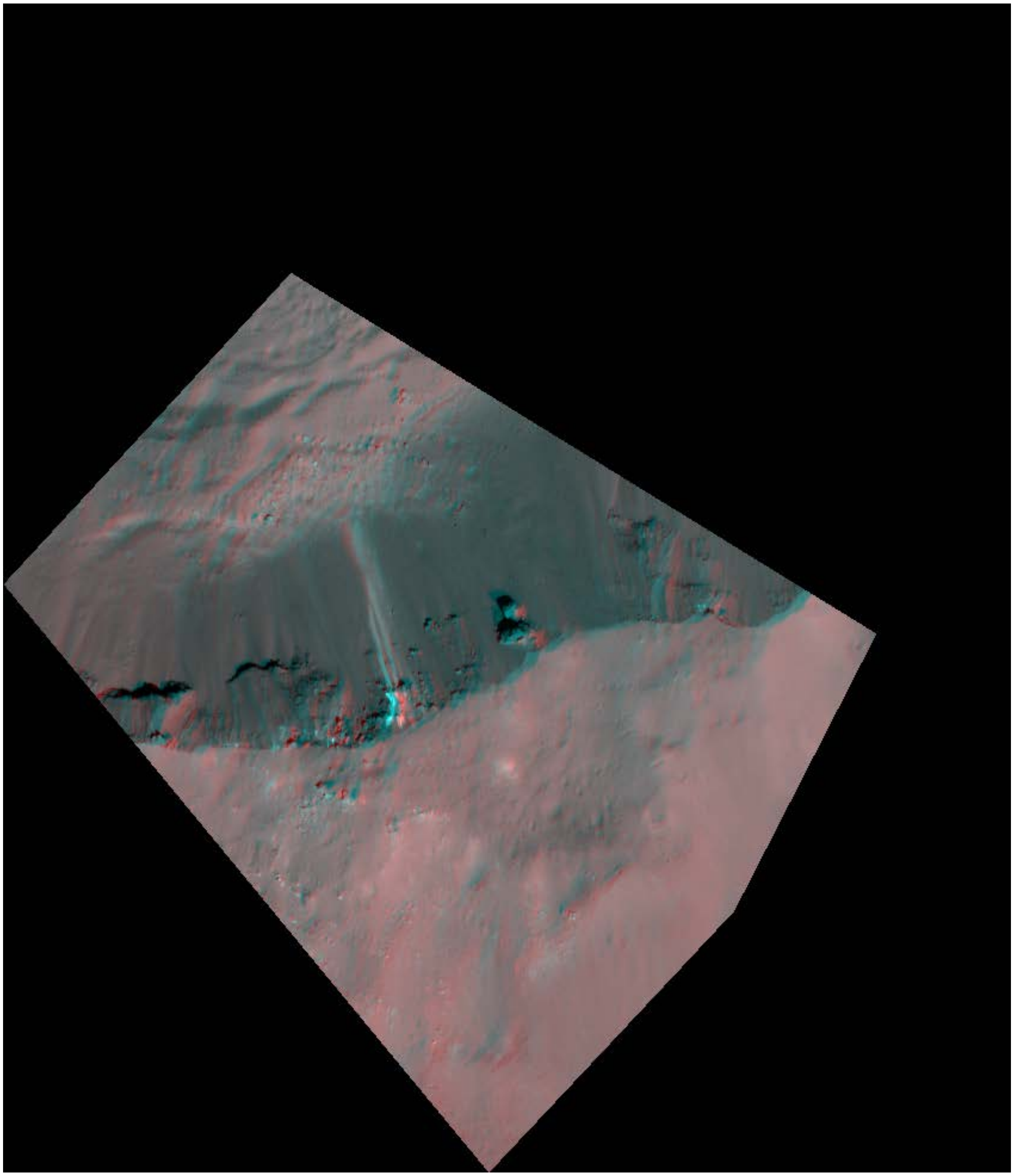

Supplementary Figure 2. Dawn XM2 stereo anaglyphs of Occator rim. Outcrops, overhangs, boulders, and small bright deposits a few 10s of meters wide are observed in several isolated exposures near the rim crest, which may be isolated outcrops of preimpact carbonate units exposed during impact. These and all stereo images in Supplementary Figures require viewing with red-blue stereo glasses.

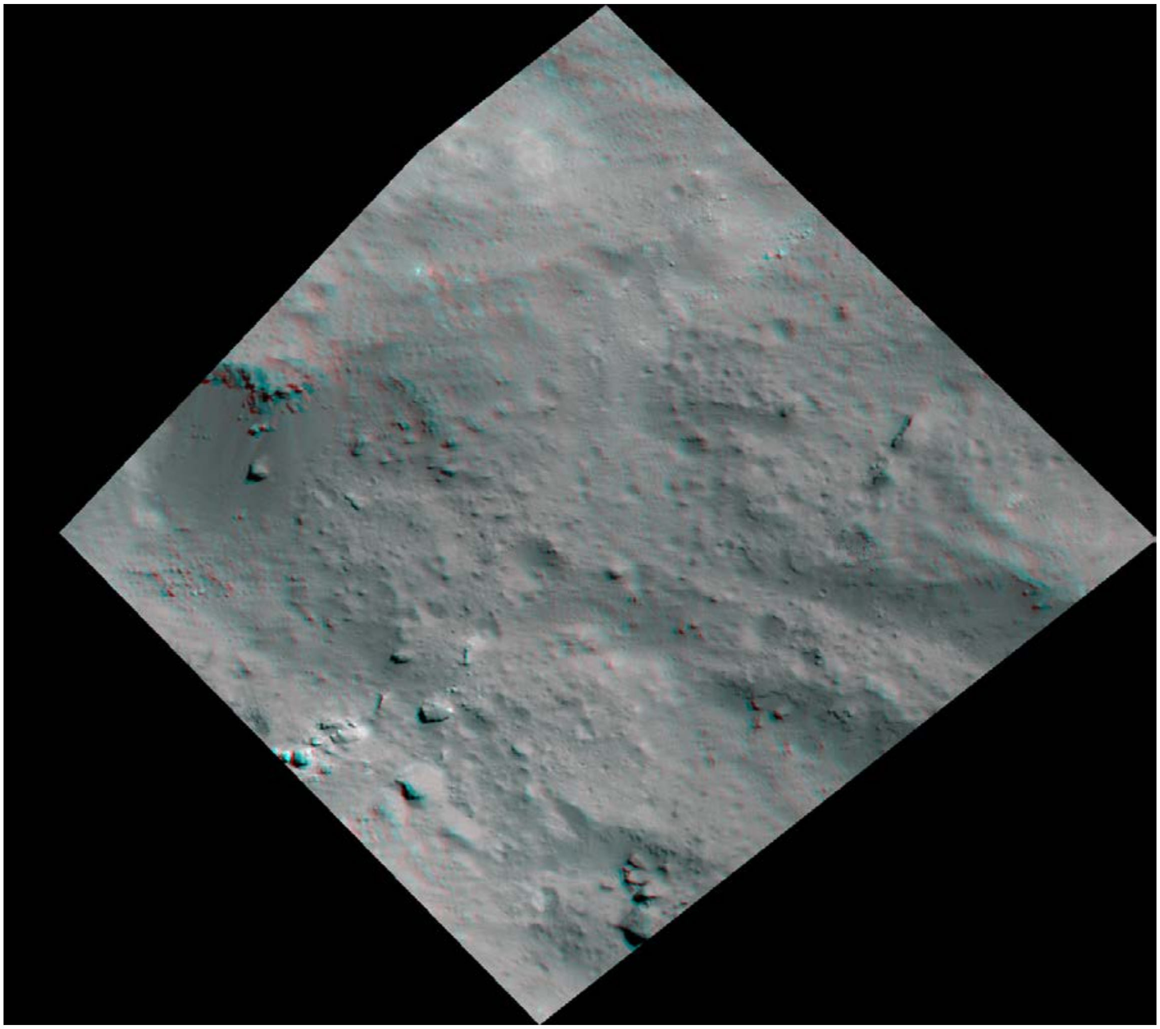

Supplementary Figure 3. Dawn XM2 stereo anaglyph Occator rim terrace.

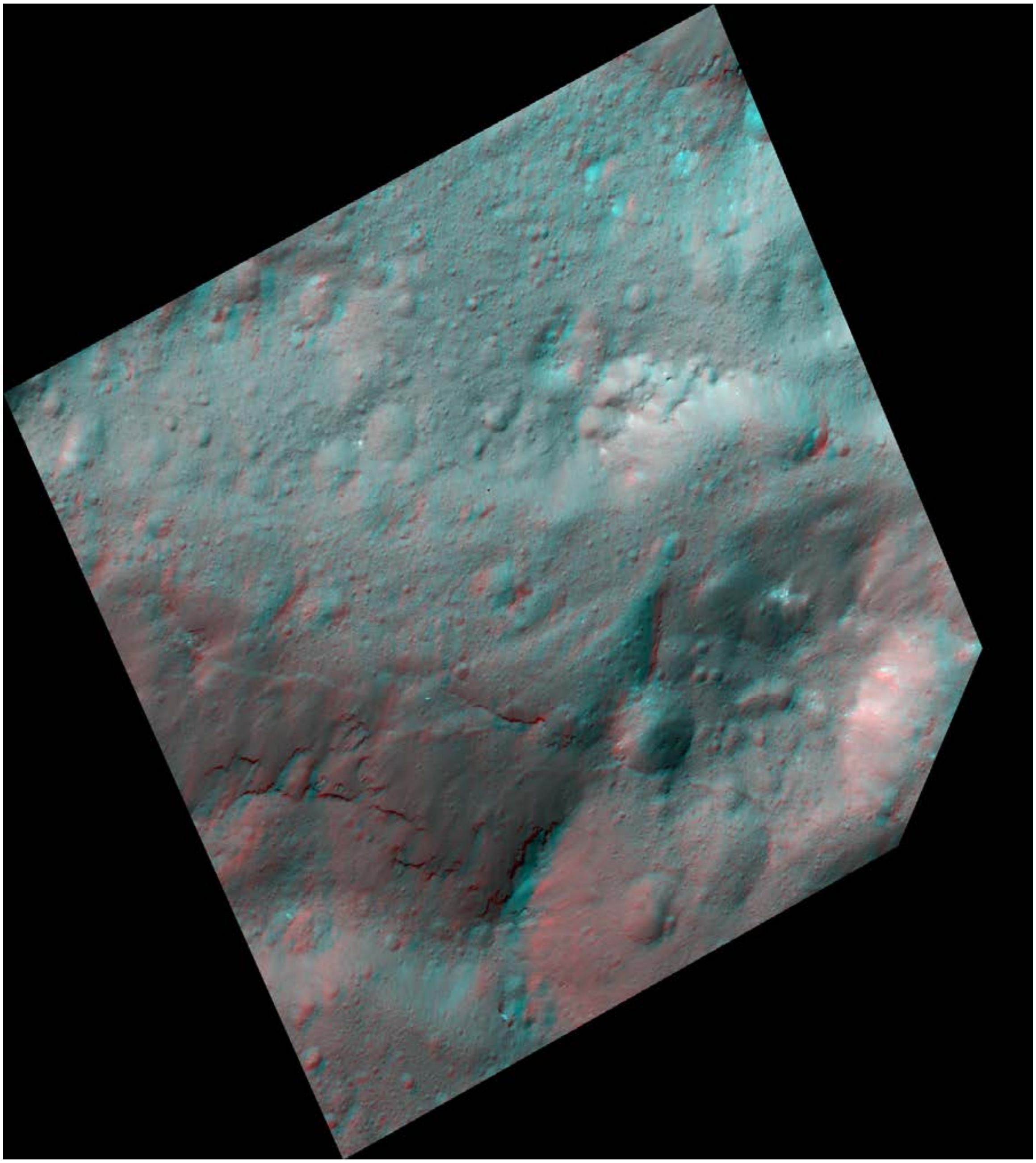

Supplementary Figure 4. Dawn XM2 stereo anaglyphs of rim and terrace morphologies at Occator crater, highlighting mantling units capping high standing terrace blocks.

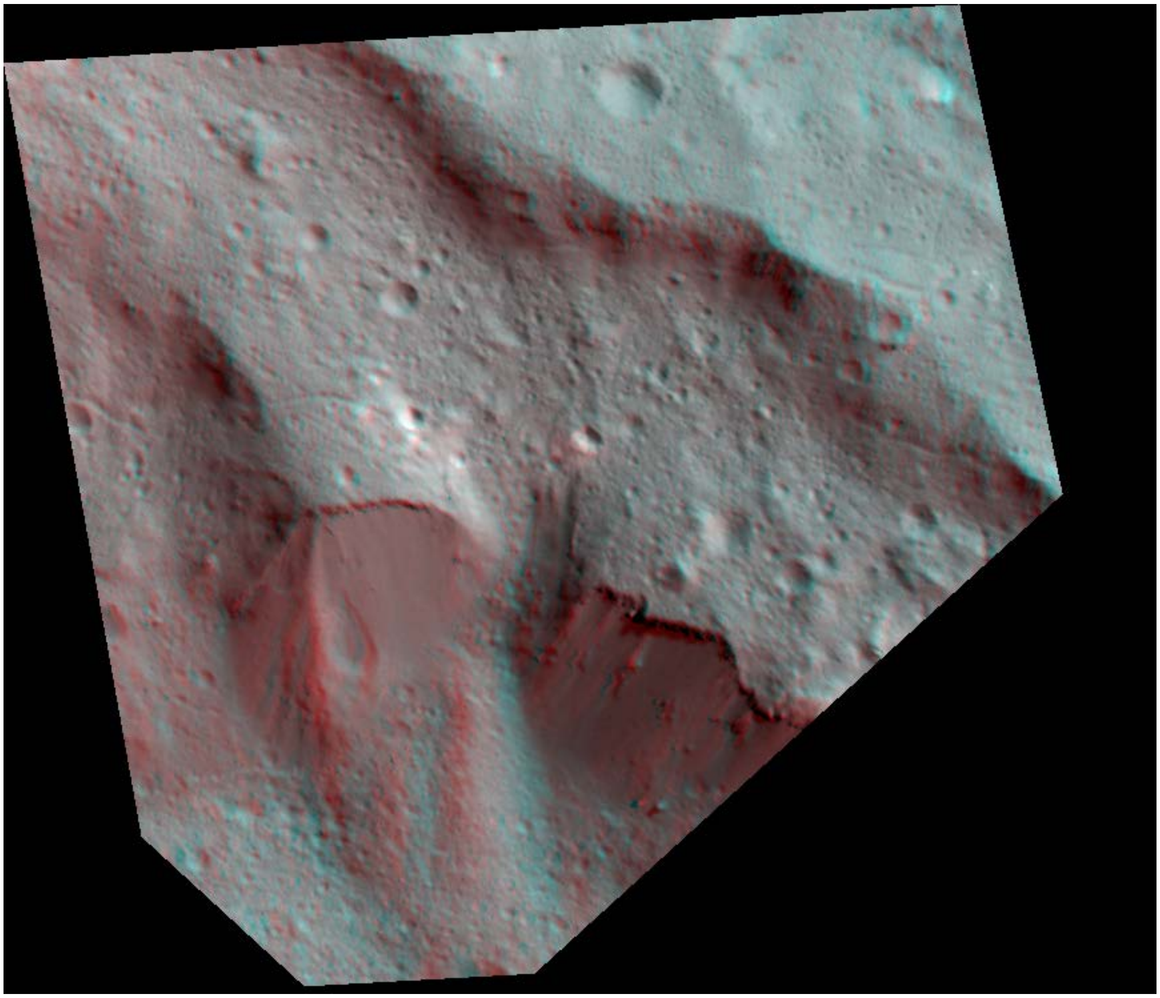

Supplementary Figure 5. Dawn XM2 stereo anaglyphs of Occator rim terrace. Scene features prominent mantling, ponded, and channelized impact melt materials draped on terrace blocks. Resistant scarp-forming unit evident at slope breaks and on flanks of terrace blocks.

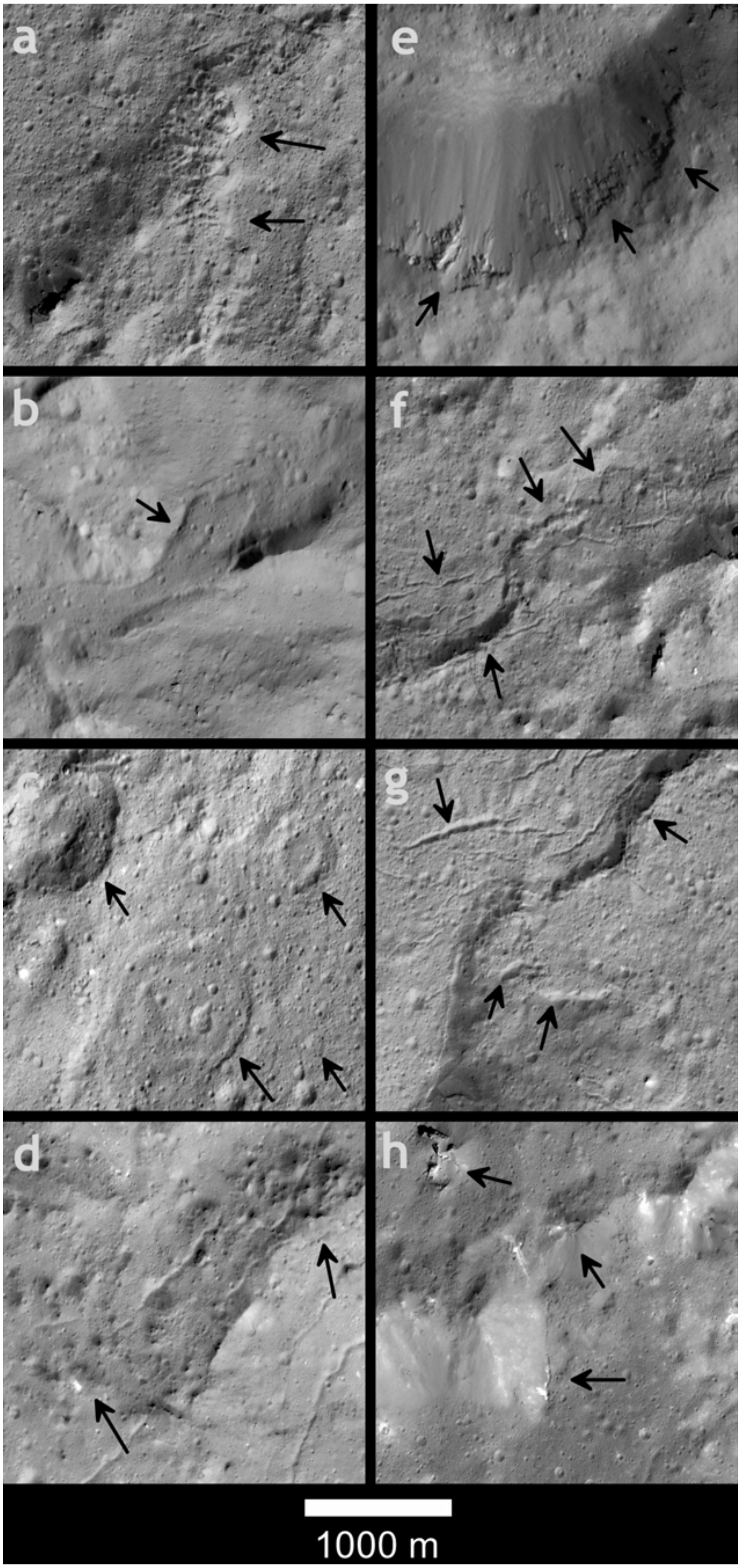
a
e
b
f
c
g
d
h
1000 m

Supplementary Figure 6. Dawn XM2 images of floor units and features in Occator. Features include: pitted materials along crest of mantled terrace ridge (a), lobate flow units (arrow) within northern crater floor units (b), shallow topographic rings (arrows) within southern lobate floor deposits (c), field of dark mounds in eastern terrace units (d), mantled terrace ridge (e), flow margins and sinuous troughs (arrows) associated with cliff and scarp (f), sinuous troughs, flow margins and pits (g), and pits and sinuous troughs on crater floor units (h).

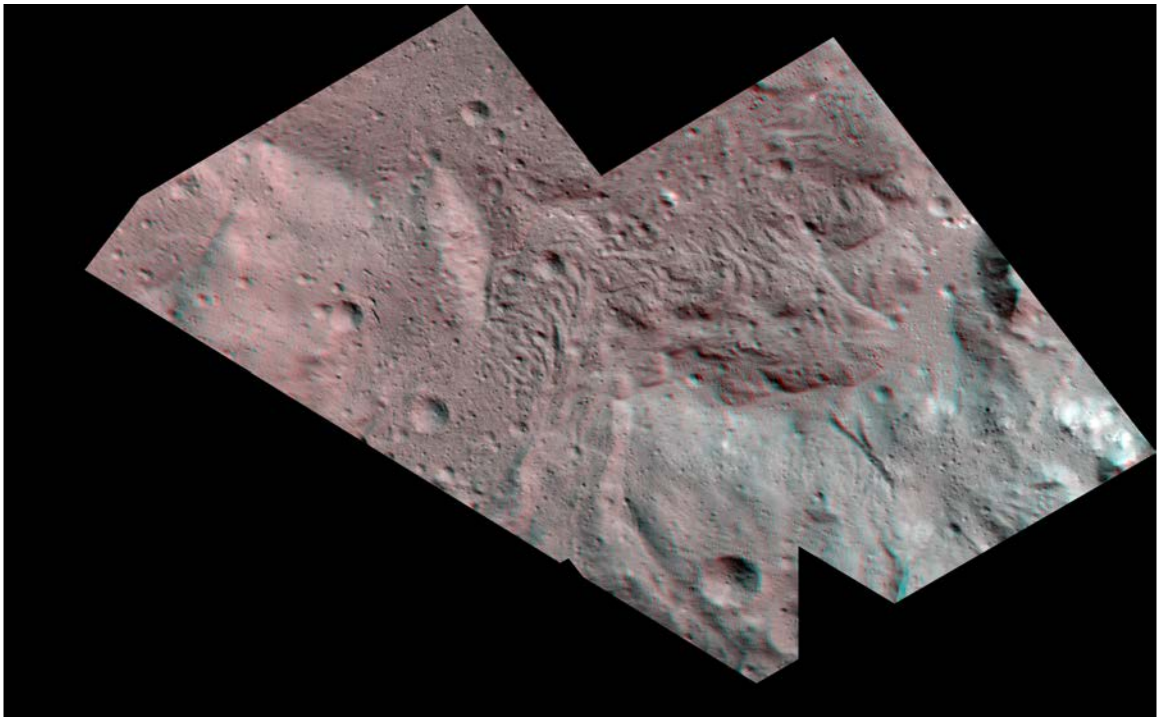

Supplementary Figure 7. Dawn XM2 stereo anaglyph of Occator floor. Mantling, ponded, and channelized impact melt materials in terrace zones and floor deposits are highlighted. Several overlapping lobate floor units overlap near center of this view.

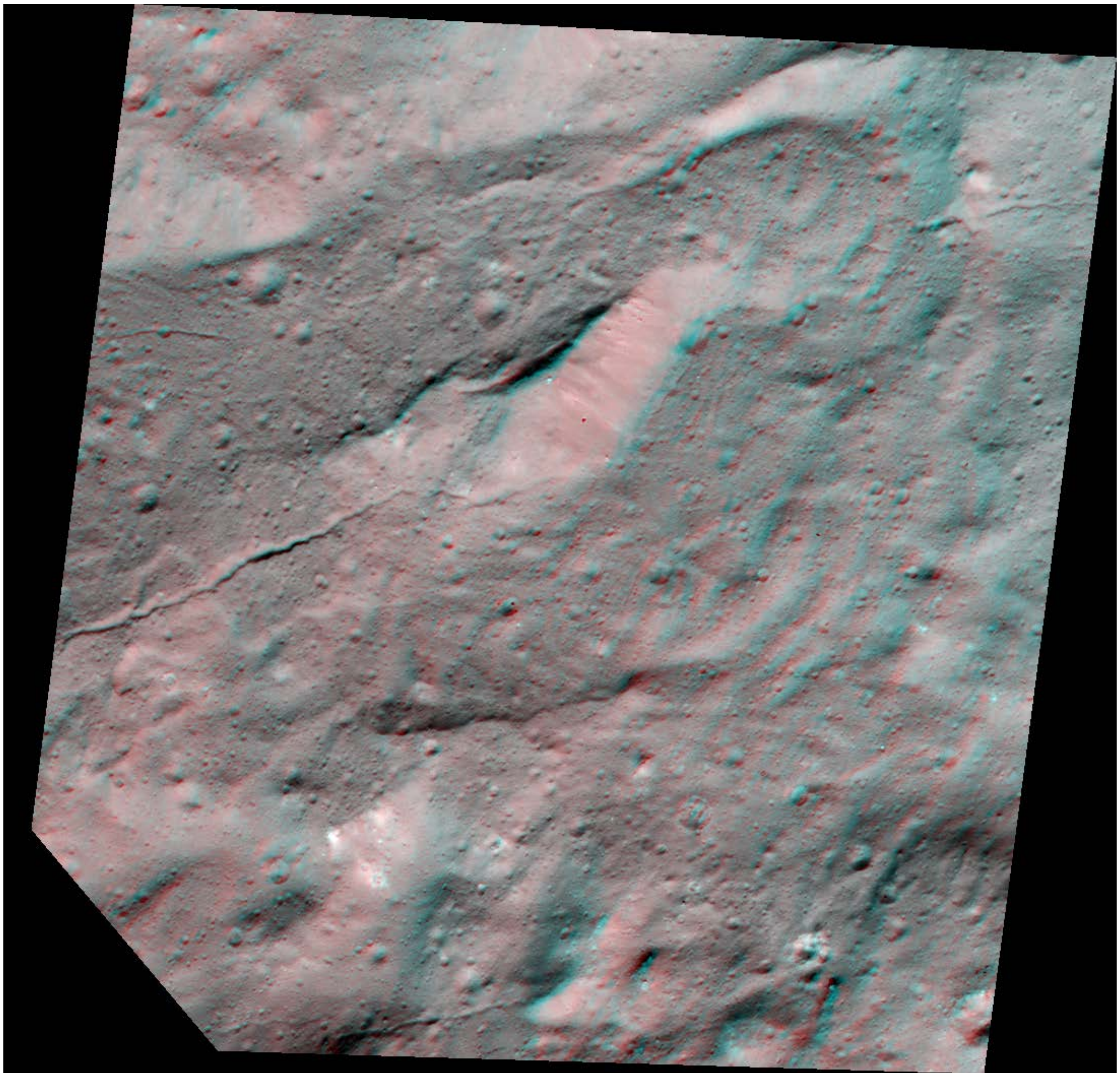

Supplementary Figure 8. Dawn XM2 stereo anaglyph of lobate flows on western Occator floor.

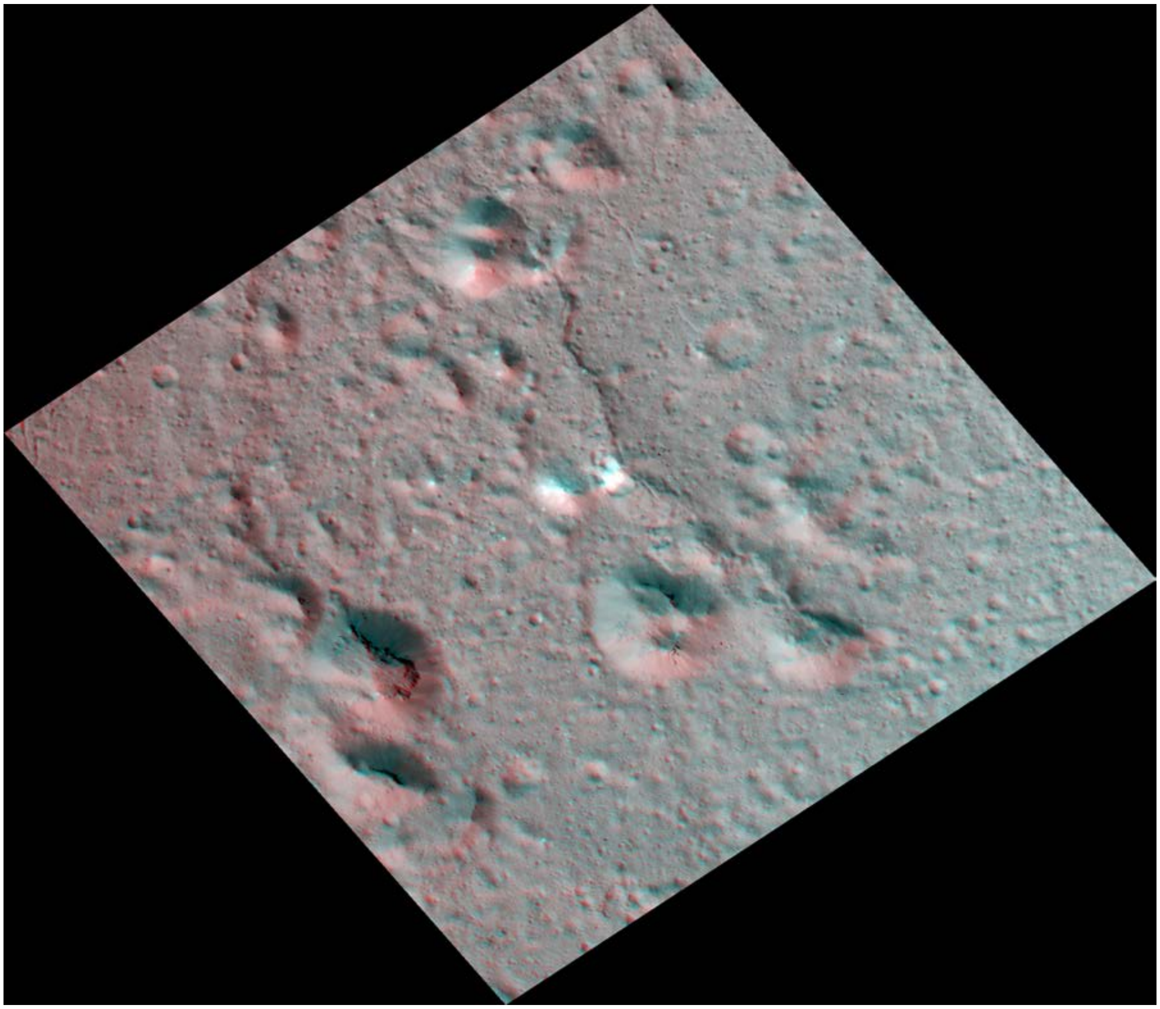

Supplementary Figure 9. Dawn XM2 stereo anaglyph of Occator floor. Scene features pits, sinuous troughs and mounds on southeastern floor deposits. See Figure 4 in main text for DTM and elevation profiles of this site.

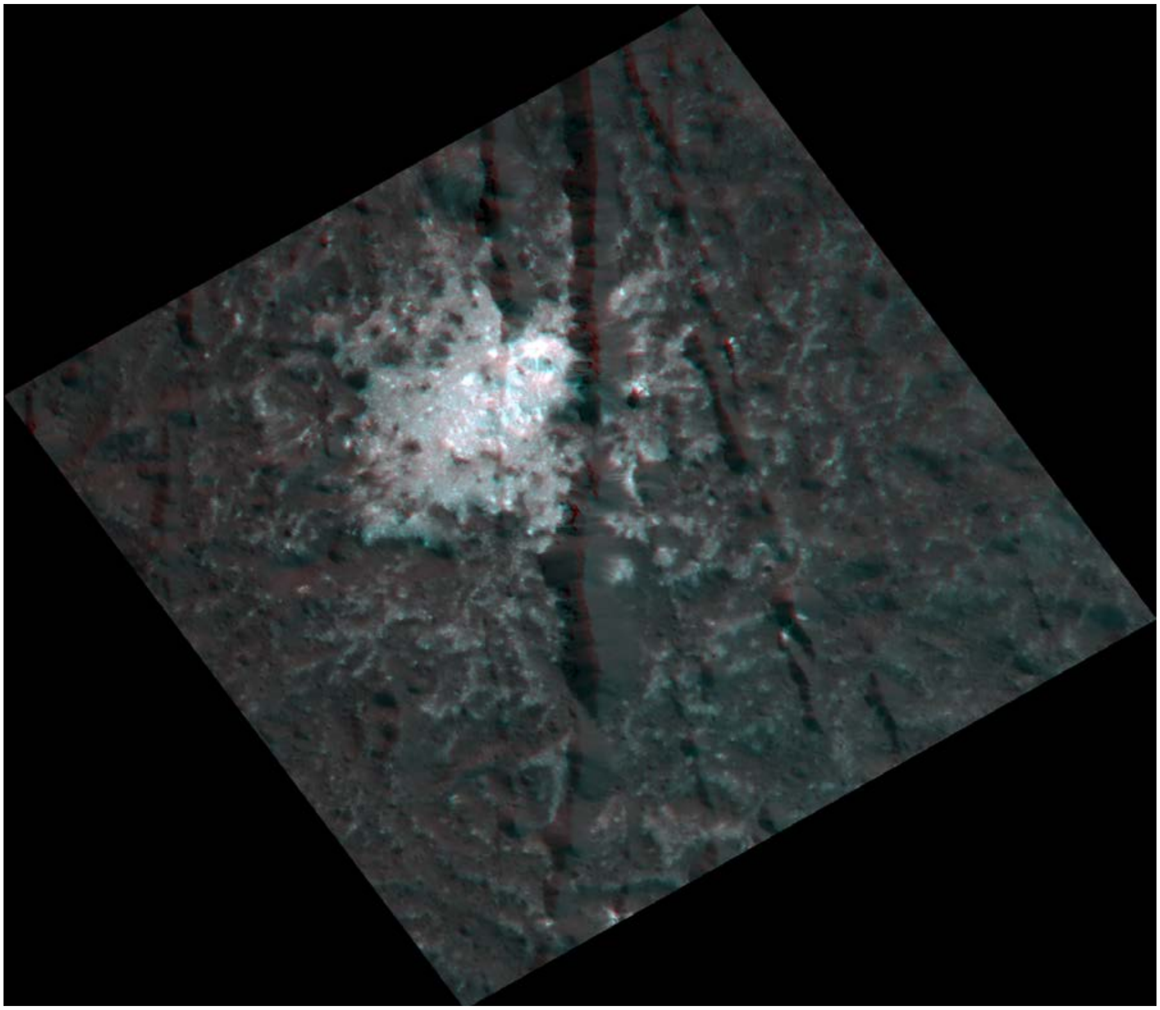

Supplementary Figure 10. Dawn XM2 stereo anaglyph of bright patch in Vinalia Faculae. Scene highlights topographic relation between the deposits (bright center e in main Figure 6) and local topography. Prominent arcuate trough cuts carbonate deposits, showing also small pits at bottom of V-shaped trough.

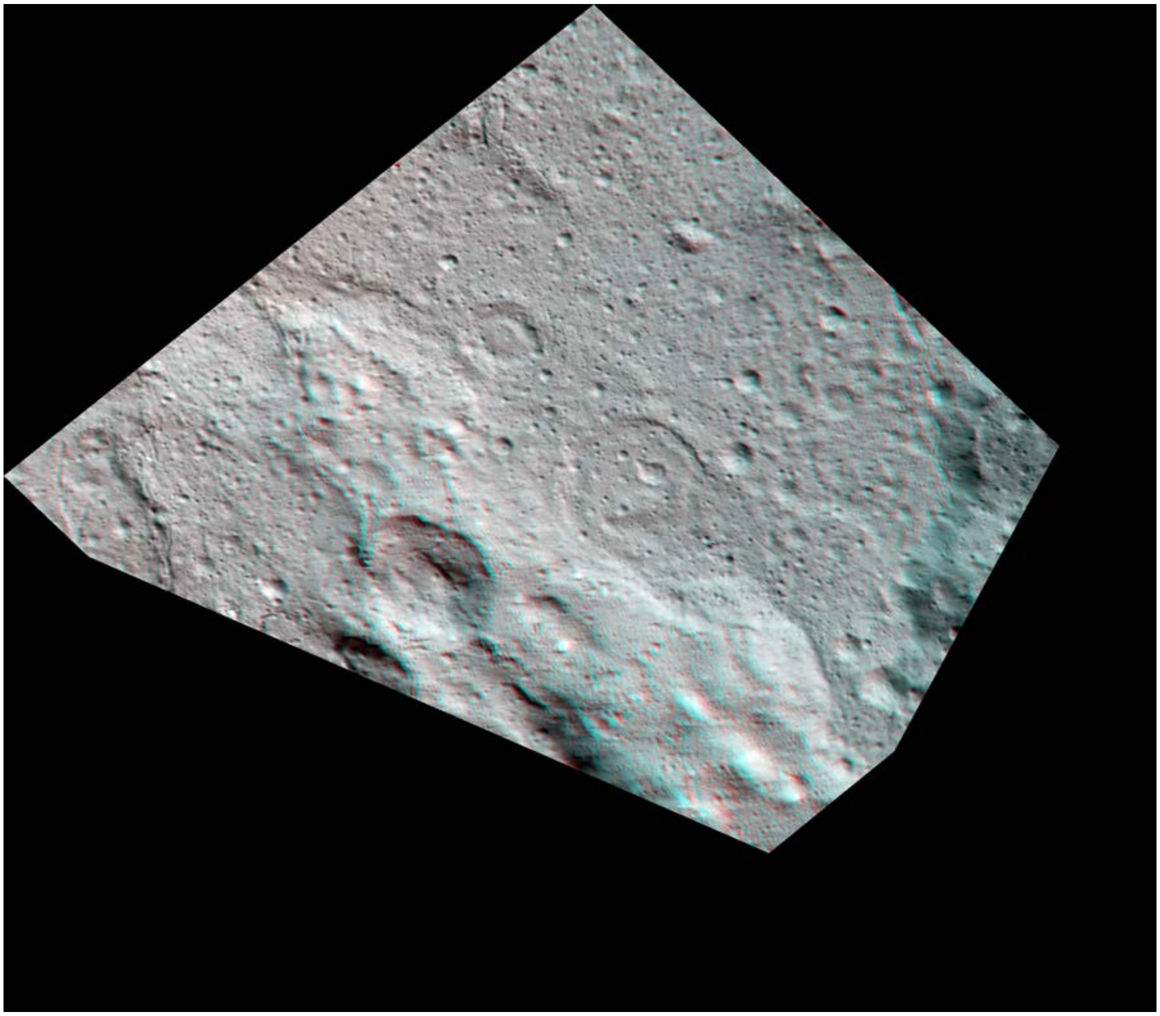

Supplementary Figure 11. Dawn XM2 stereo anaglyph of shallow rings in lobate floor deposits (LFD). Also visible is a small patch of fractured material near top and a deeper conical ring structure at lower left.

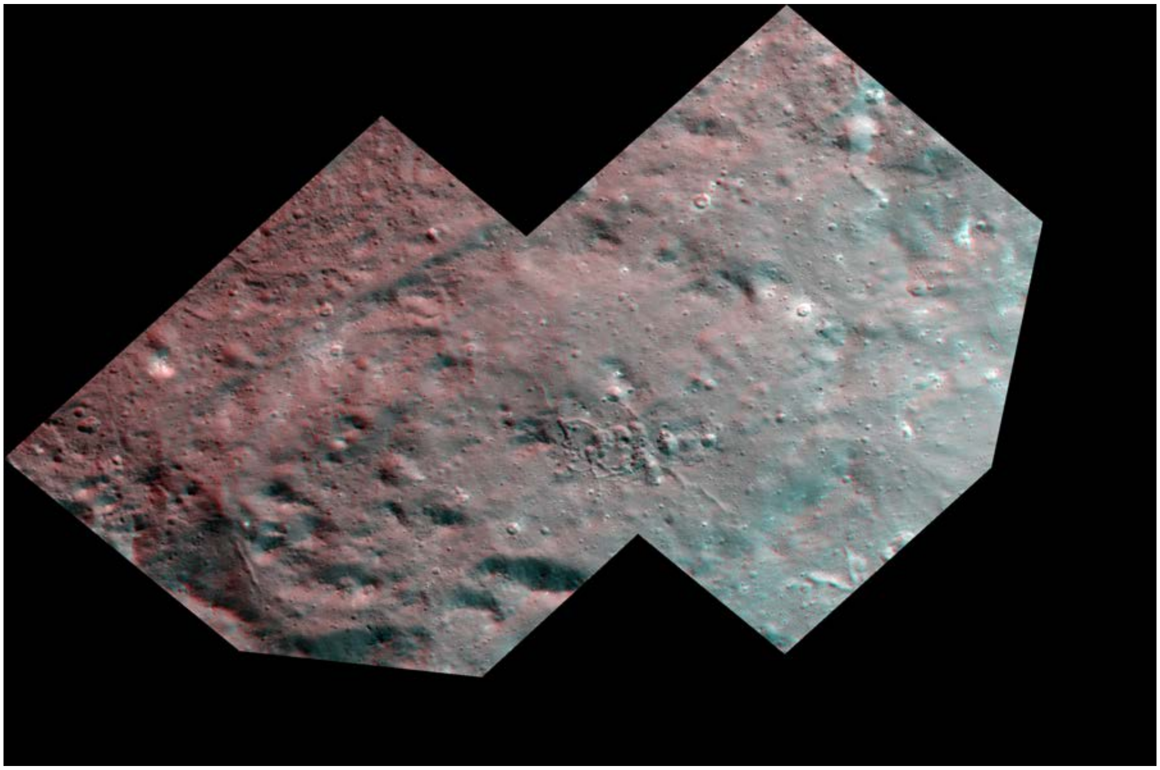

Supplementary Figure 12. Dawn XM2 stereo anaglyph of prominent pit cluster on floor of Occator. See Figure 5 in main text for DTM and elevation profiles of this site.

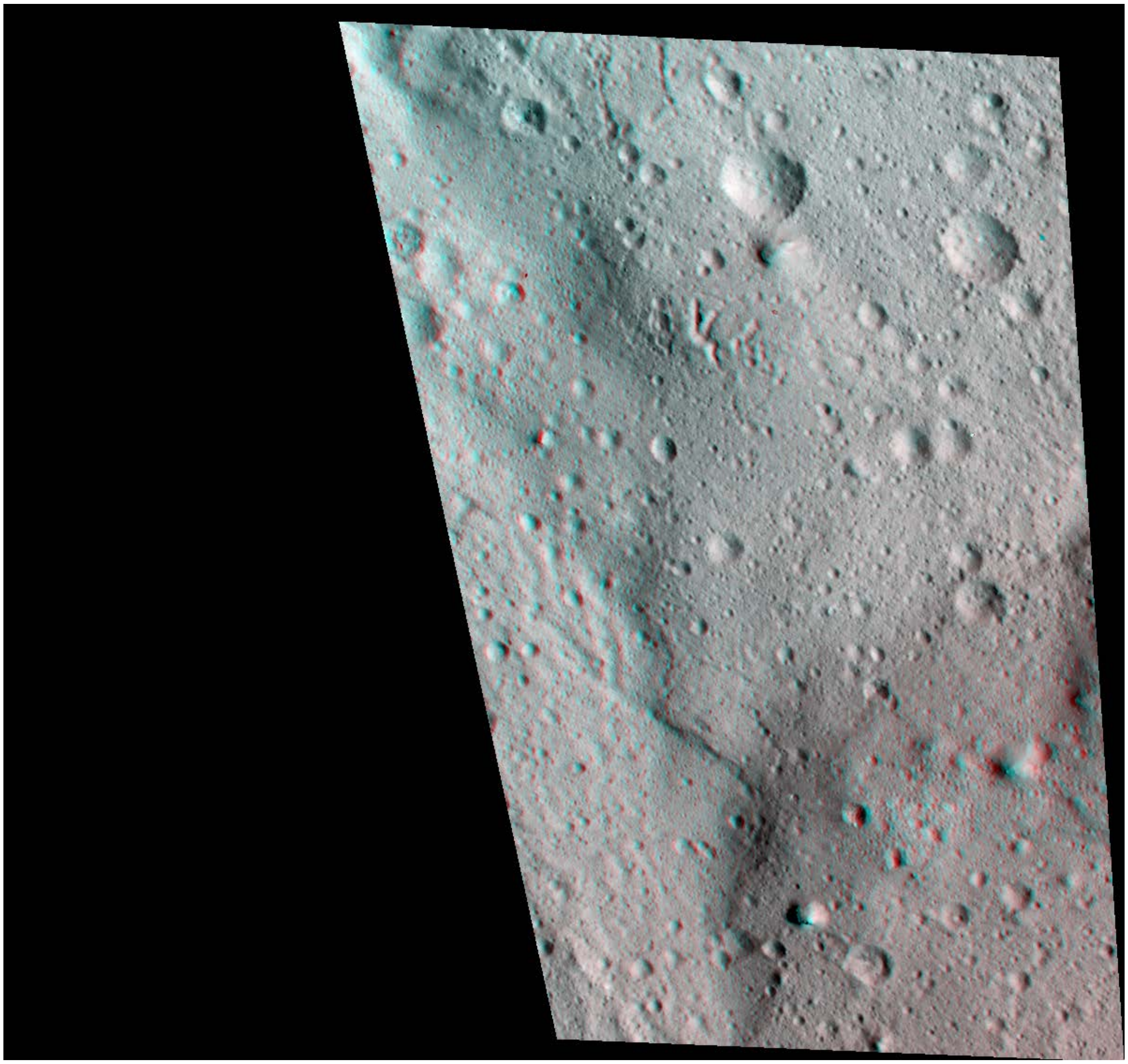

Supplementary Figure 13. Dawn XM2 stereo anaglyph of northern floor of Occator. Scene features sigmoidal pits and conical mounds within in hummocky and ridged floor units. Sigmoidal depressions are ~40 m deep.

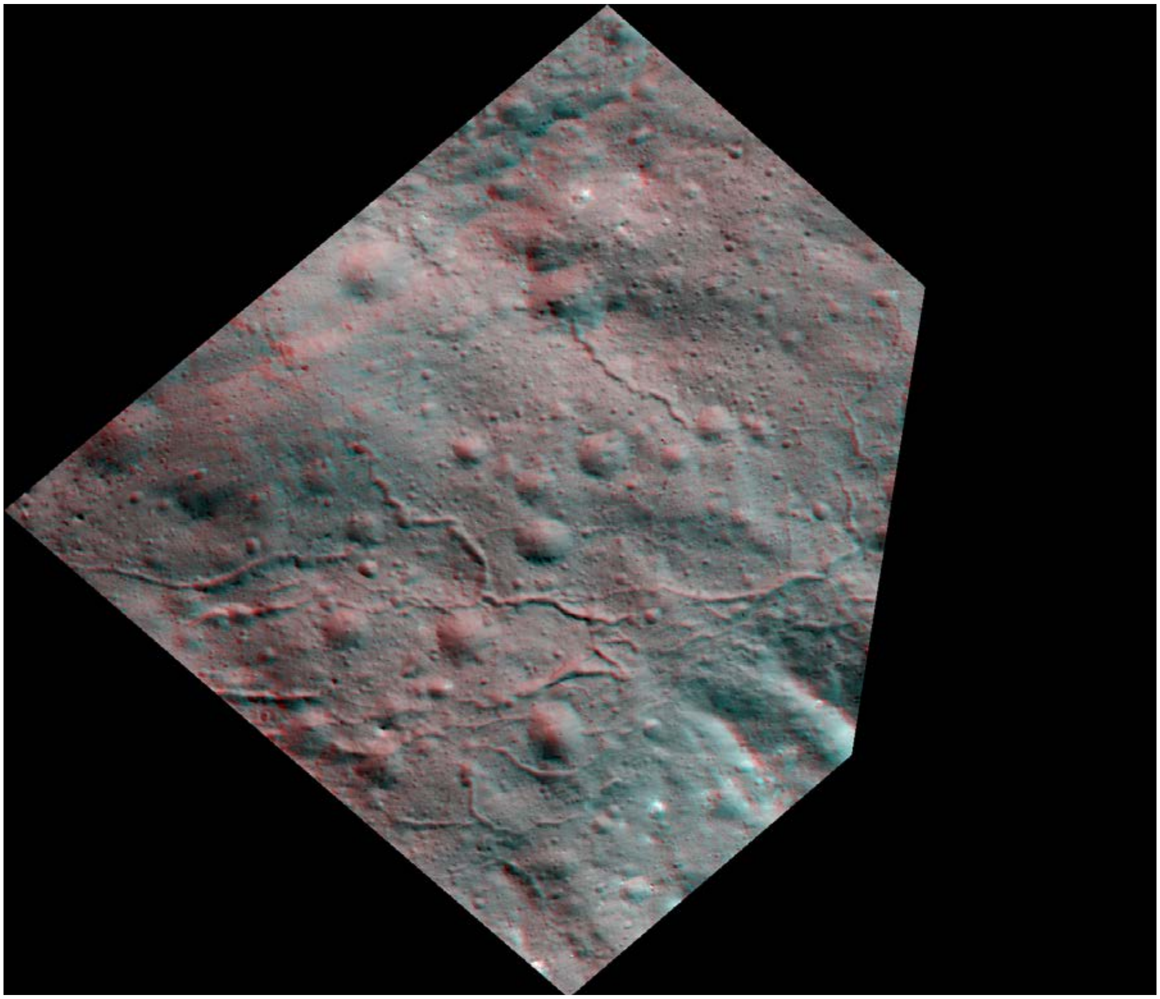

Supplementary Figure 14. Dawn XM2 stereo anaglyph of eastern Occator floor. Scene highlights narrow sinuous trough crossing large ridge structure within the eastern terrace zone.

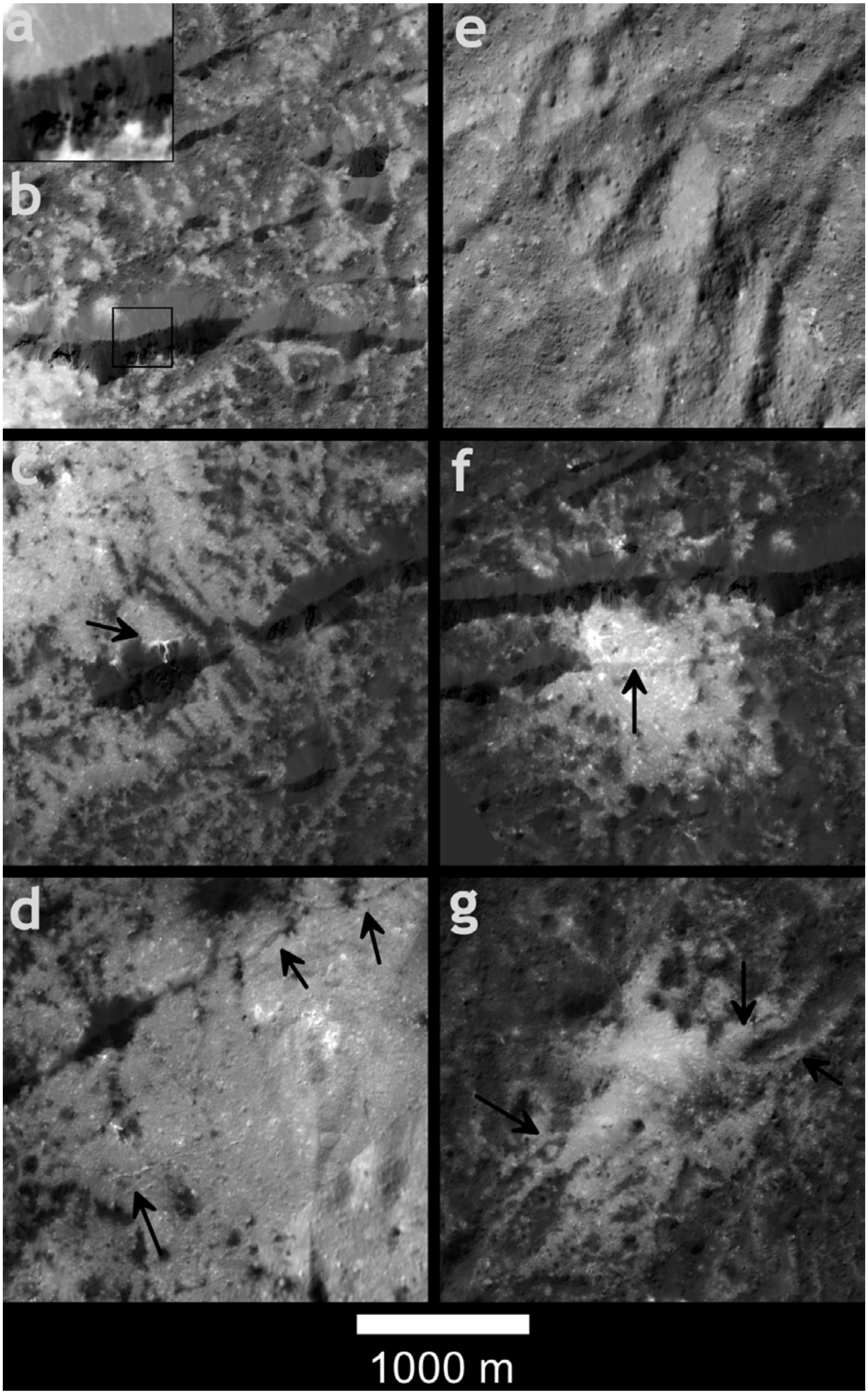
a
b
c
d
e
f
g
1000 m

Supplementary Figure 15. Dawn XM2 mosaics of carbonate deposits in Vinalia Facula. Features include: (a, b) pinch and swell arcuate trough segments in western Vinalia Faculae (VF) (inset (a) shows small pits at bottom of trough), (c) bright ribbon of carbonate material down steep slope of arcuate trough formed by mass wasting, (d) extensive carbonate materials in eastern VF mantling older arcuate trough (upper arrows) but cut by post deposition sinuous trough (lower arrow), (e) ridge and trough morphology characteristic of lobate floor deposits in eastern Occator that form basal surface on which VF formed, (f) concentration of carbonate materials in western VF mantling earlier formed linear fracture (arrow), (g) concentration of carbonate materials in western VF mantling embaying ridge and trough morphology of lobate floor material (arrows).

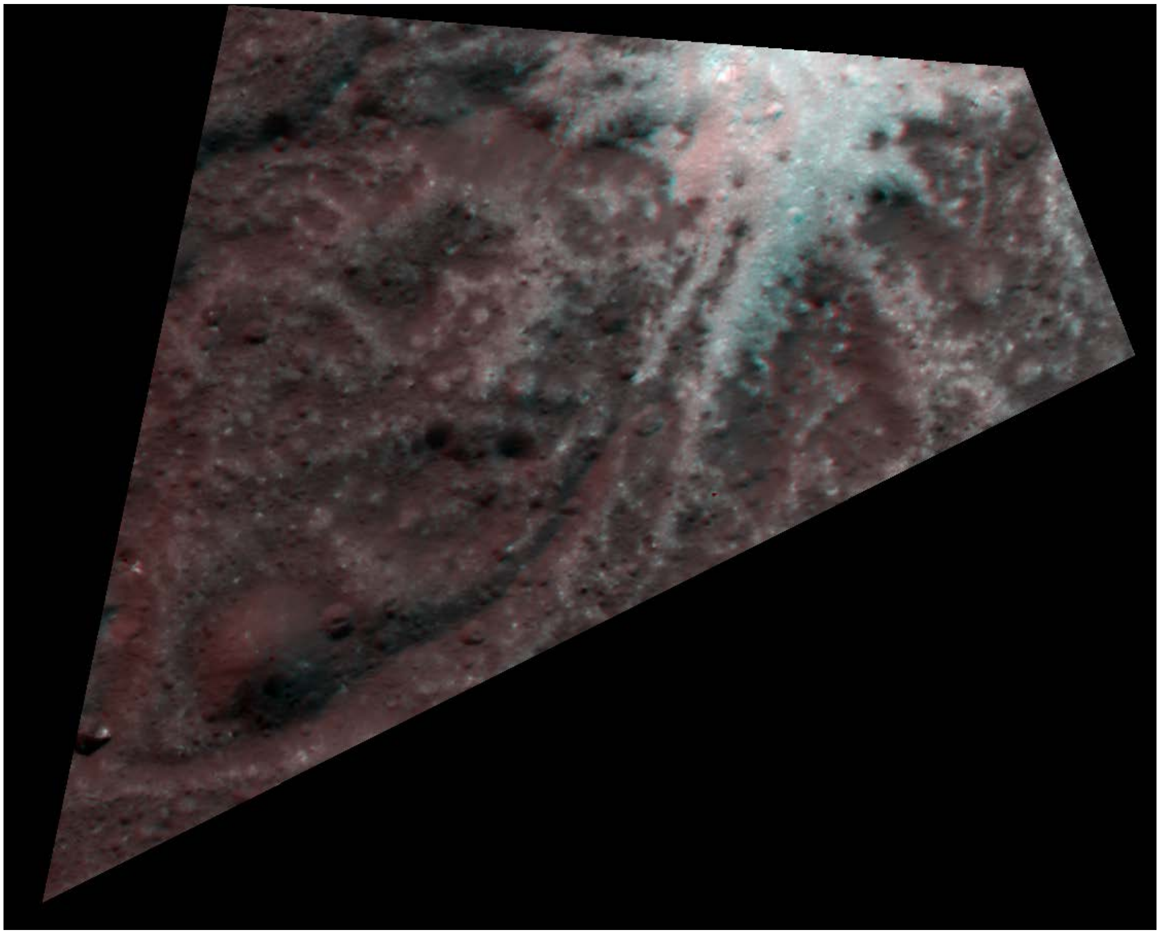

Supplementary Figure 16. Dawn XM2 stereo anaglyph of bright center at Vinalia Faculae. Scene shows bright center [c] (from Figure 6 in main text) in Vinalia Faculae, showing topographic relation between the deposits and local topography. Also evident are the numerous small pits on the bright deposit, interpreted as impact craters.

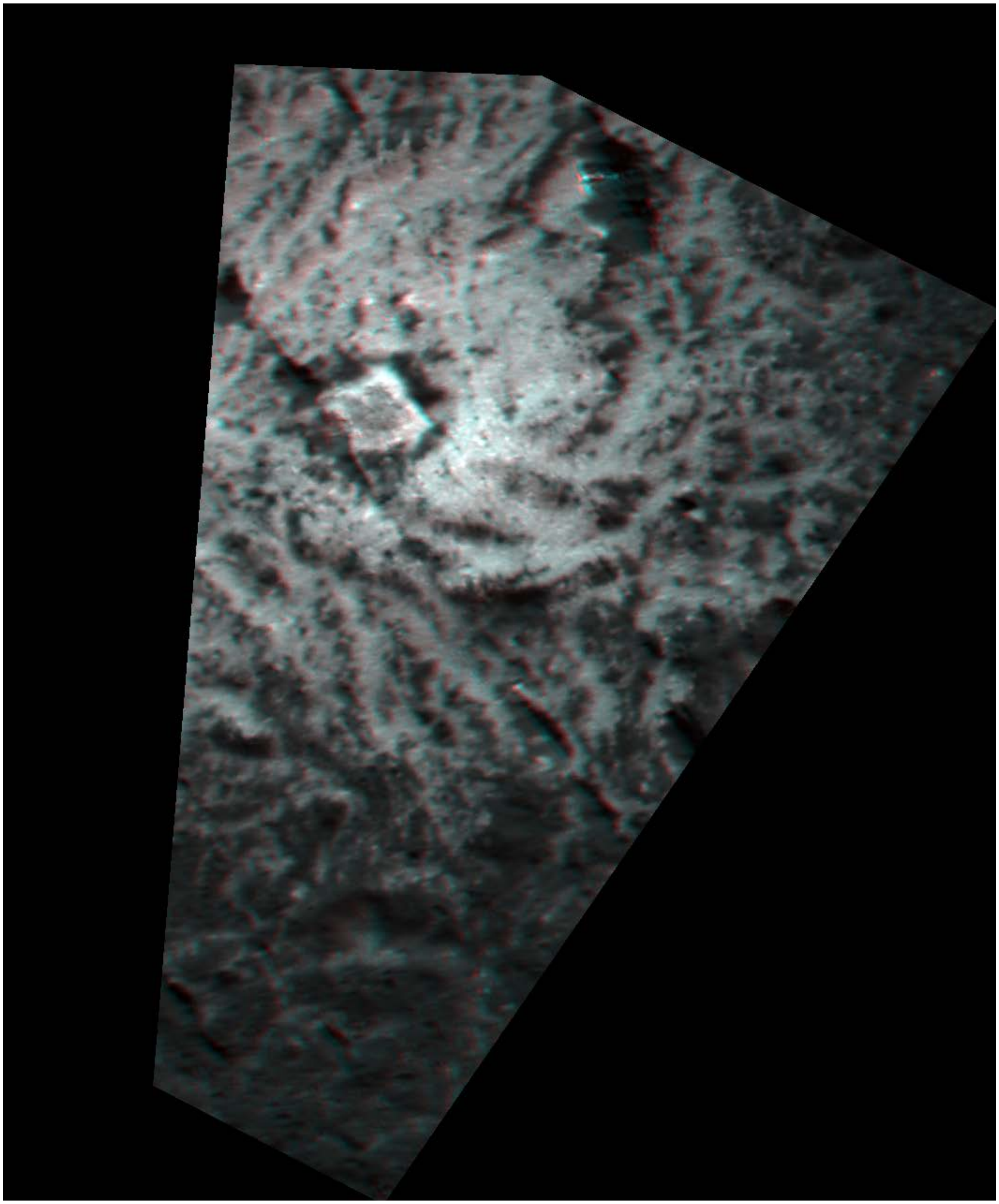

Supplementary Figure 17. Dawn XM2 stereo anaglyph of bright center in Vinalia Faculae. Scene highlights topographic relation between the deposits and local topography in Vinalia Faculae bright center [g] (in main text Figure 6). Also shown are dark ring and numerous small pits on the bright deposit, interpreted as impact craters.

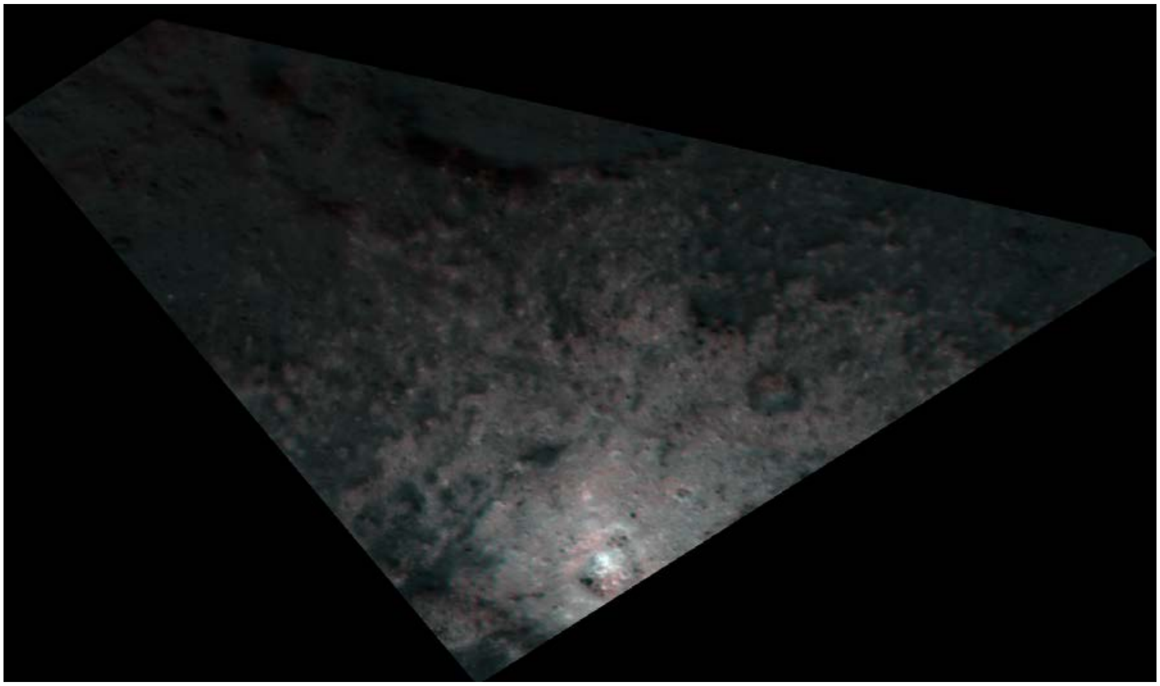

Supplementary Figure 18. Dawn XM2 stereo anaglyph of small dome in Vinalia Facula. Apparent constructional dome (at bottom center) is in VF bright center [i] (in main text Figure 6).

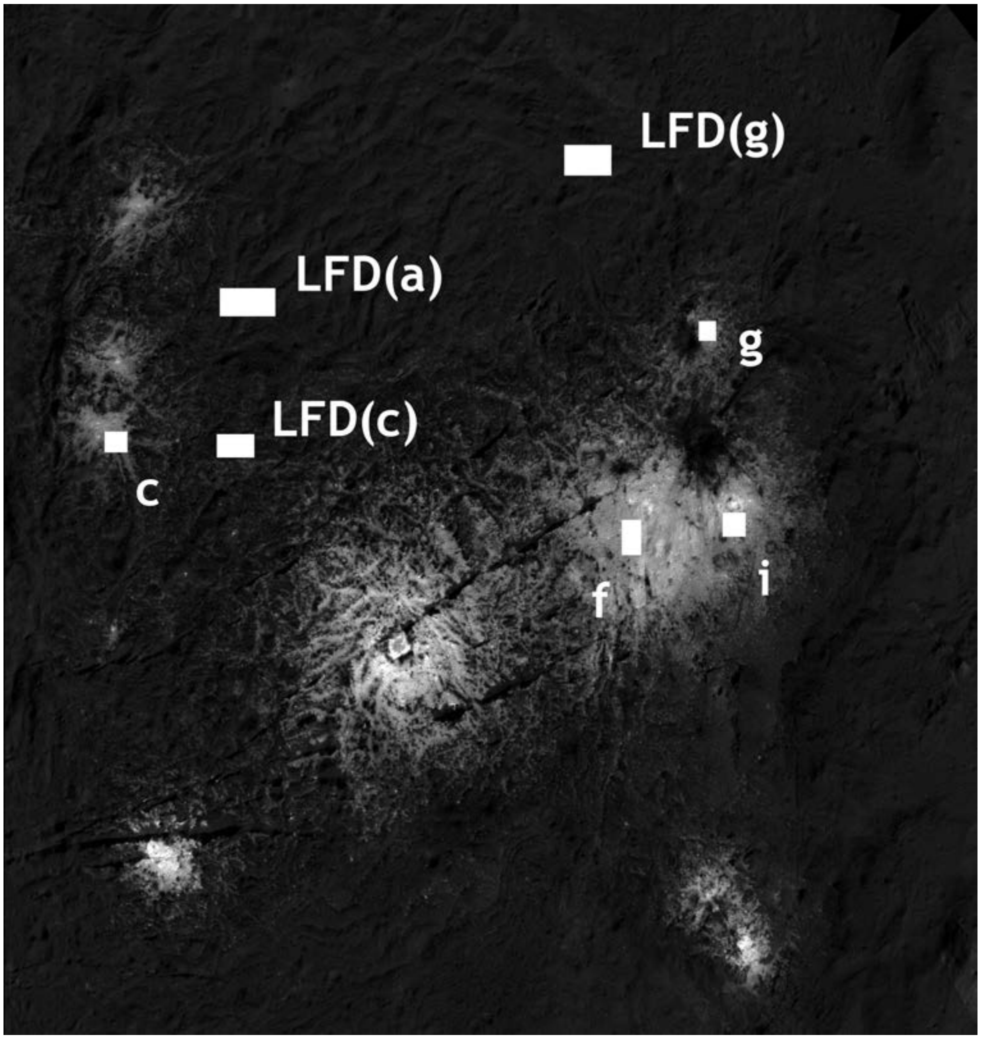

Supplementary Figure 19. Crater counting sites within and proximal to Vinalia Facula. Bright boxes indicate locations of counting areas plotted in Supplementary Figure 21. Labels (e.g., c, f, g, i) indicate bright centers of carbonate deposition identified in main text Figure 6 and described in text. LFD are sites within unmodified lobate floor deposits.

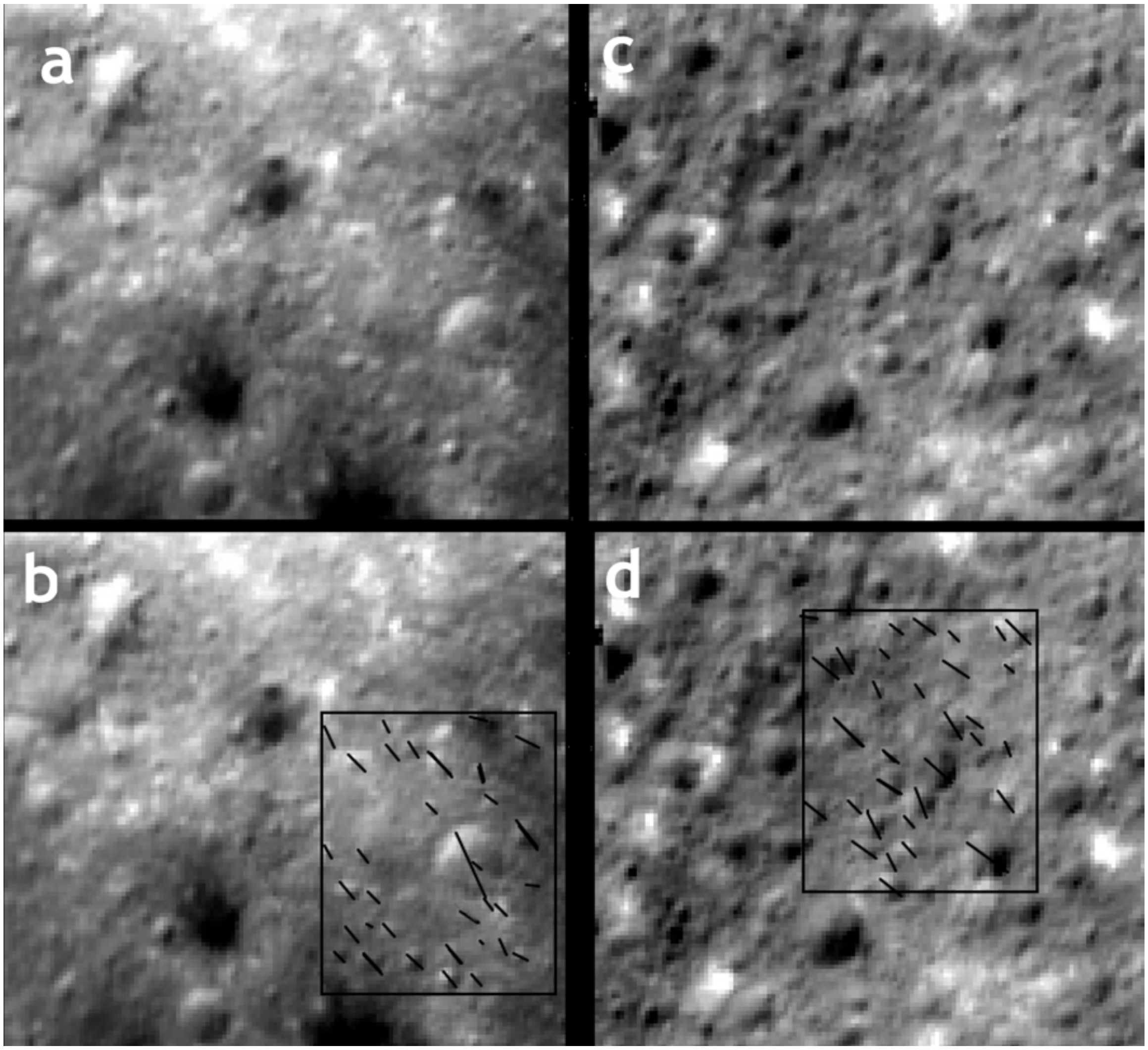

Supplementary Figure 20. Two counting areas for age determination within Vinalia Facula. Sites are for bright center [c] and LFD adjacent to [c] located in Suppl. Figure 19. Counting sites are carbonate deposits (a, b) and lobate floor materials (c, d), with square counting areas and measured craters marked in (b) and (d). Scene widths are 440 m in width; counting areas in boxes are ~200 m across.

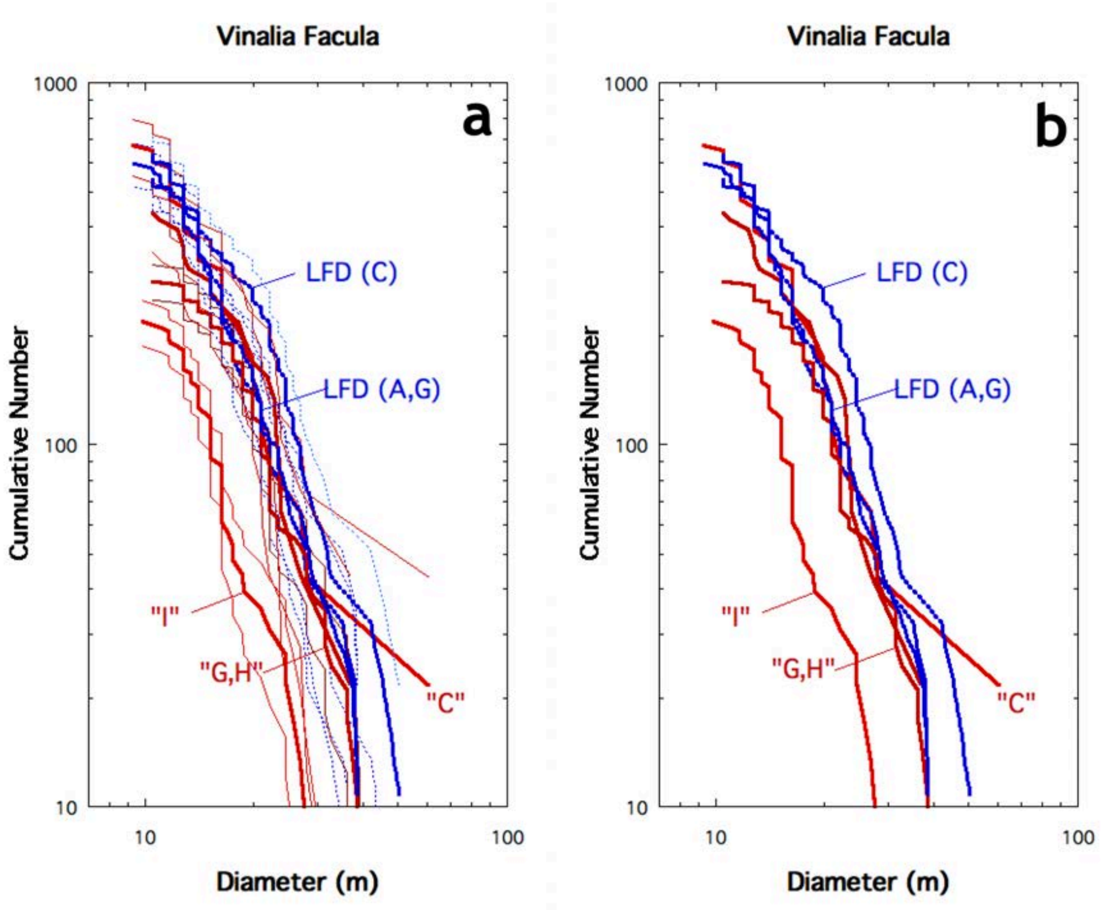

Supplementary Figure 21. Crater counts for sites within and proximal to Vinalia Facula. Counts are shown with (a) formal uncertainties (a) and without (b) for clarity. Cumulative crater plot for crater densities on lobate floor deposits (LFD) adjacent to VF (blue lines, LFD), and 4 of the VF major centers (red lines; ["c", "g,h" and "i"). Site locations are shown in Supplementary Figure 19. The right-hand graph shows the same plot without the standard uncertainties (thin lines in left-hand graph) for clarity. Derived asteroid-flux based age estimates are ~8 myr (LFD (c)), ~4 myr (LFD (a, g) and "g, h", "c") and ~1.7 myr ("i"). Formal age uncertainties are ~0.5 myr, except "i" which is ~0.2 myr, but real uncertainties are likely larger due to the very small counting areas (<0.1 km^2). Thus all sites except "i" are statistically indistinguishable in apparent crater retention age, and all sites including "i" likely have larger uncertainties than the formal uncertainties shown in panel (a).

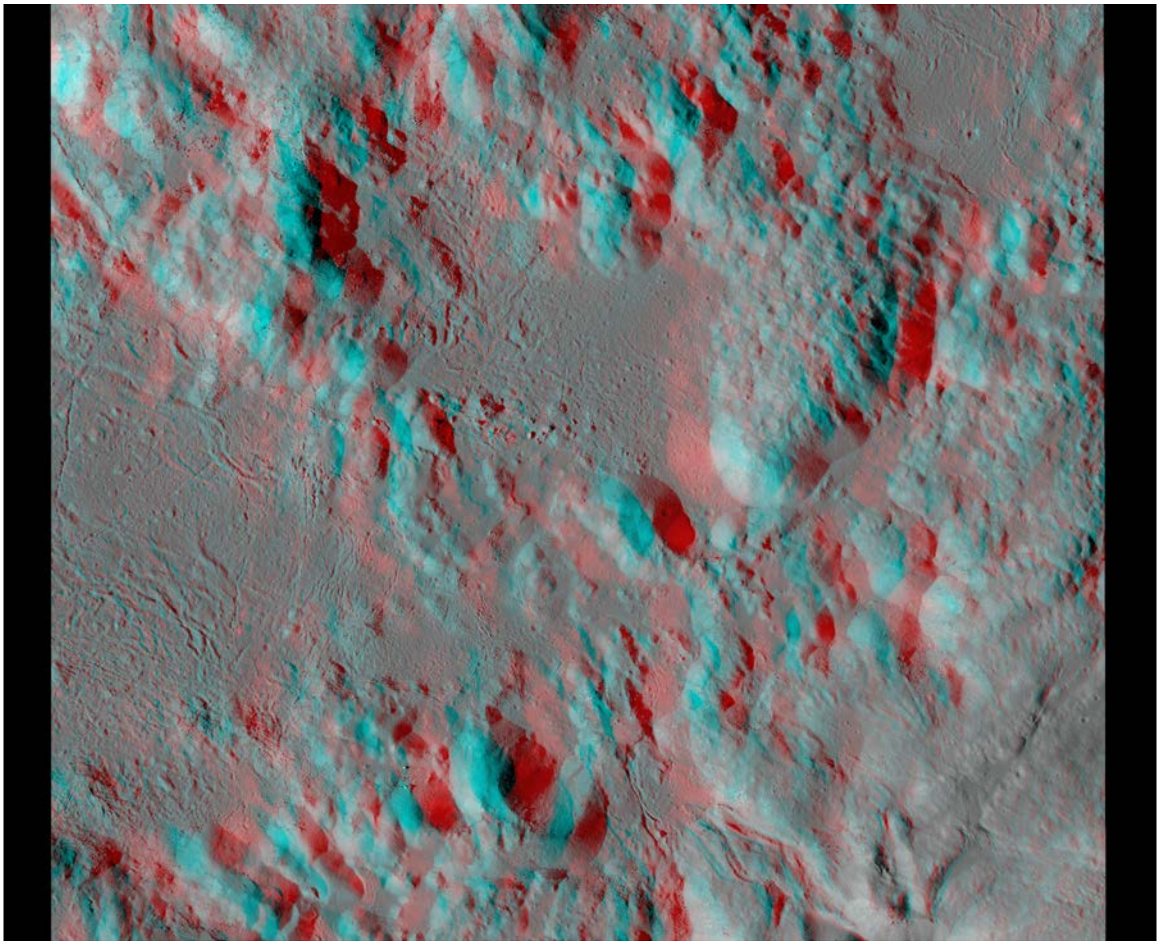

Supplementary Figure 22. LROC stereo anaglyph of smooth and ridged southern floor deposits of 71-km-wide lunar crater Jackson, showing level units at different elevations and embayed in narrow valleys.

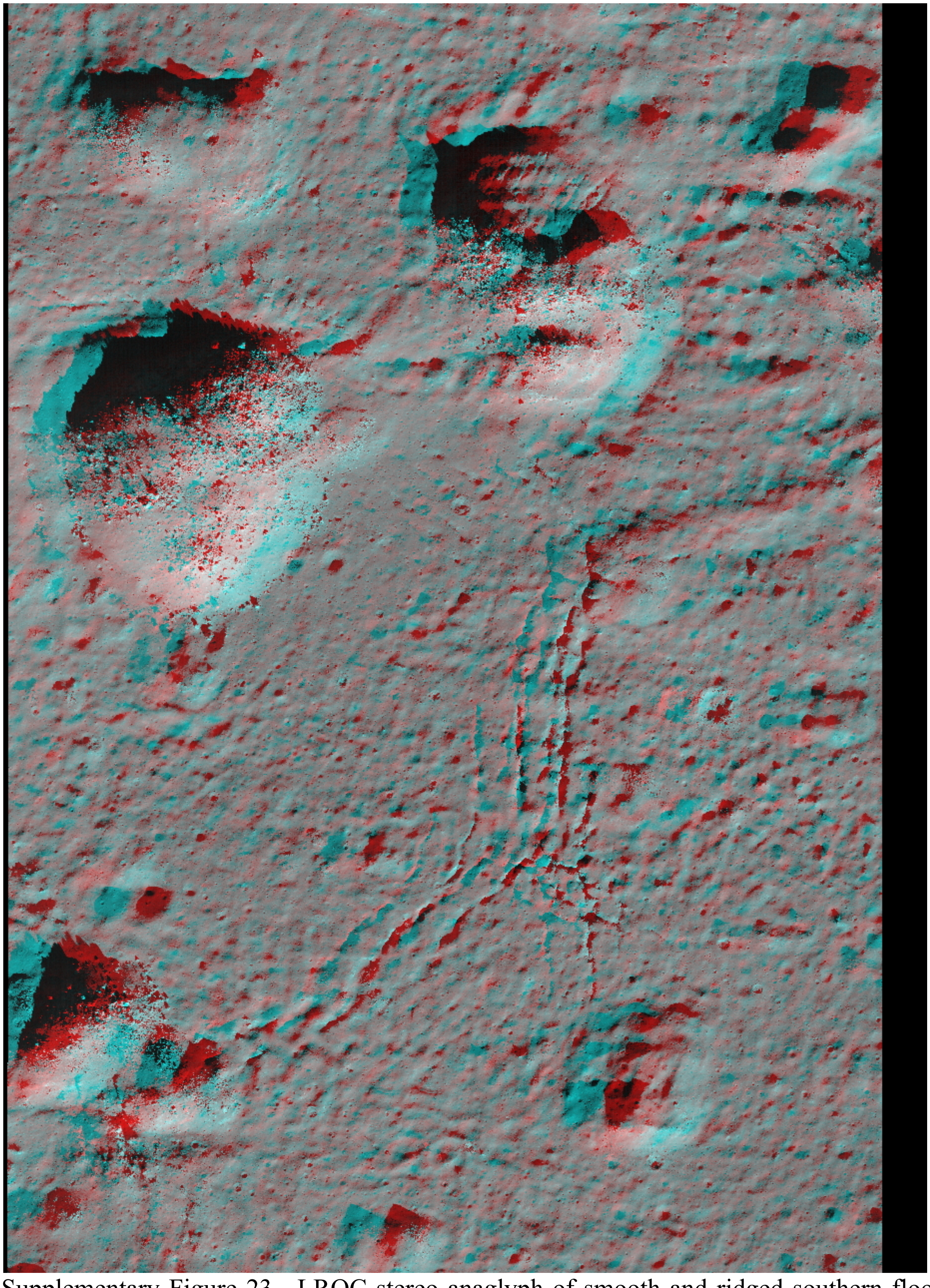

Supplementary Figure 23. LROC stereo anaglyph of smooth and ridged southern floor deposits of 52-km-wide lunar crater Anaxagoras, showing low hummocky melt plains, including portions at higher topographic levels, as well as rounded and flat-topped floor mounds.

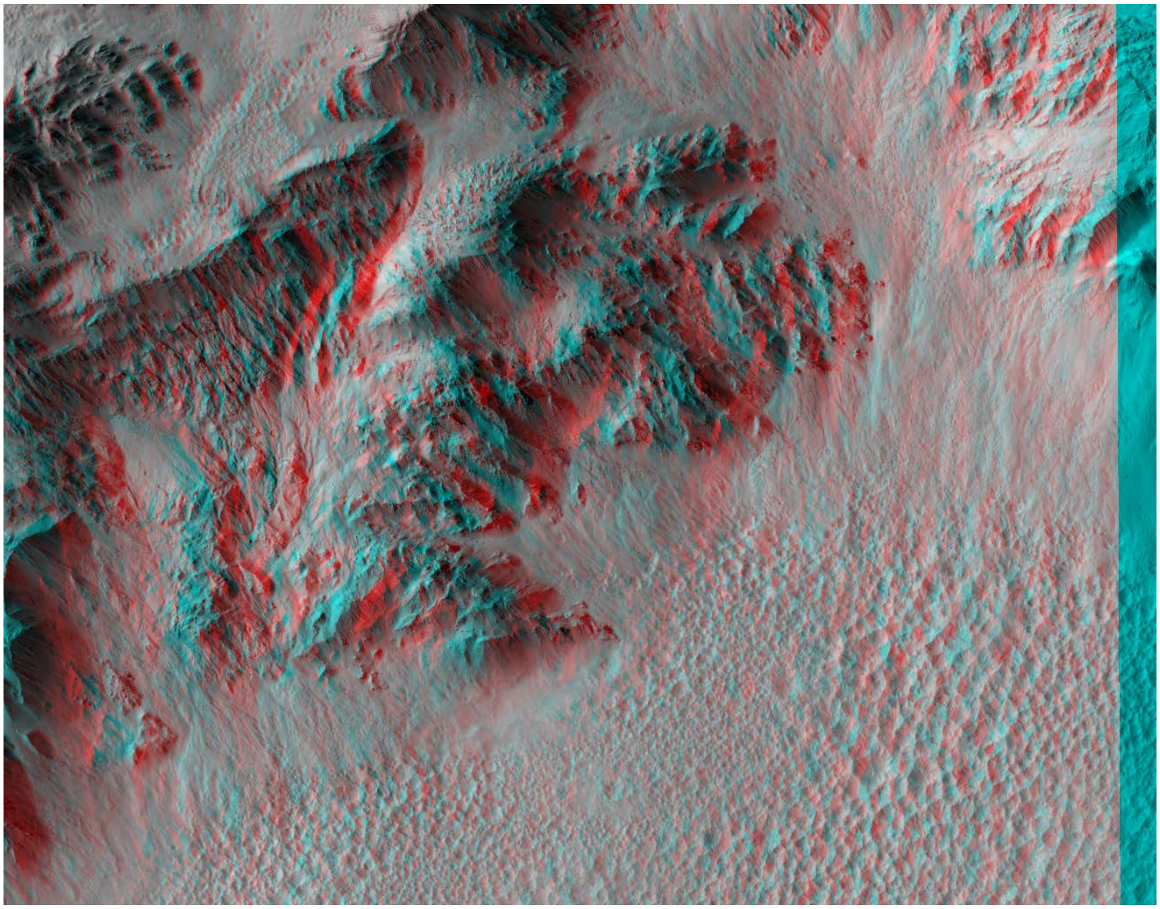

Supplementary Figure 24. HiRise stereo anaglyphs (top and bottom) of pitted and fractured southern and northern floor deposits of martian crater Tooting. Melt material 'draining' off central peak material is also evident.

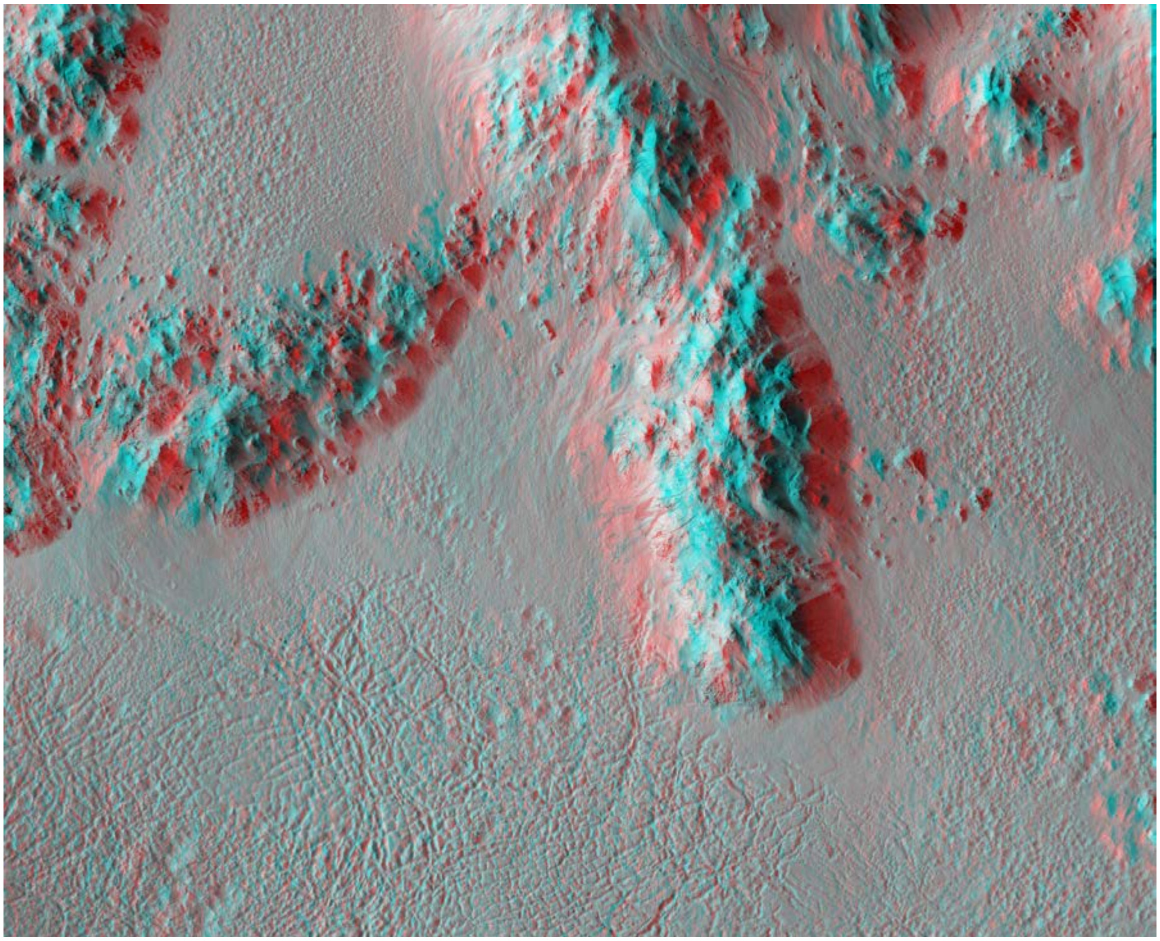

Supplementary Figure 25. HiRise stereo anaglyphs (top and bottom) of pitted and fractured southern and northern floor deposits of martian crater Tooting. Melt material ‘draining’ off central peak material is also evident.

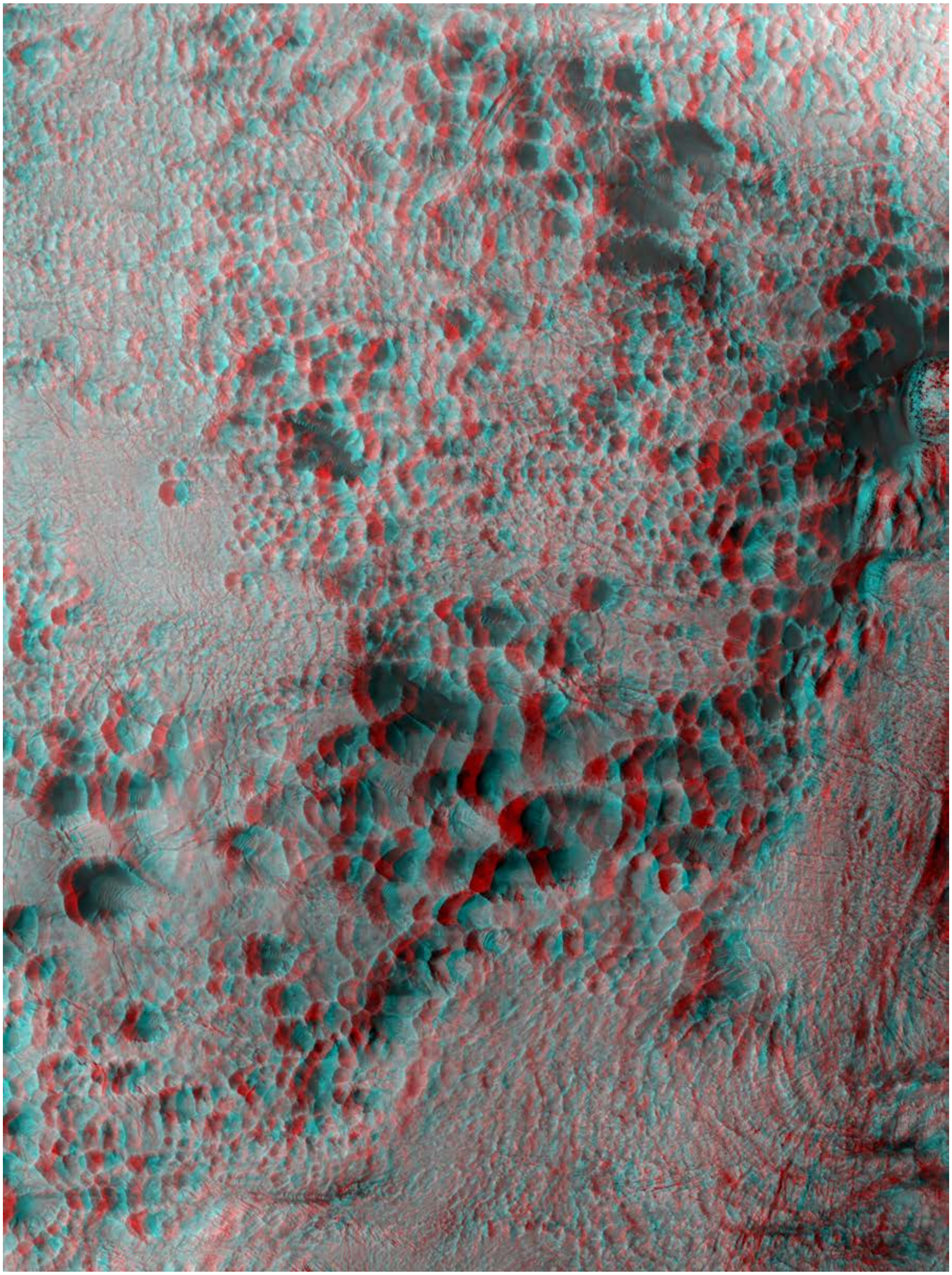

Supplementary Figure 26. HiRise stereo anaglyph of densely pitted and fractured southern and northern floor deposits of 59-km-wide martian crater Mojave.
